# Indoor Environmental Exposures and Exacerbation of Asthma: An Update to the 2000 Review by the Institute of Medicine

**DOI:** 10.1289/ehp.1307922

**Published:** 2014-10-10

**Authors:** Watcharoot Kanchongkittiphon, Mark J. Mendell, Jonathan M. Gaffin, Grace Wang, Wanda Phipatanakul

**Affiliations:** 1 Division of Allergy and Immunology, Boston Children’s Hospital, Boston, Massachusetts, USA; 2 Harvard Medical School, Boston, Massachusetts, USA; 3 Department of Pediatrics, Ramathibodi Hospital, Mahidol University, Bangkok, Thailand; 4 Indoor Air Quality Program, California Department of Public Health, Richmond, California, USA; 5 Indoor Environment Group, Lawrence Berkeley National Laboratory, Berkeley, California, USA; 6 American Institutes for Research, San Mateo, California, USA.; * These authors contributed equally to this work.

## Abstract

Background: Previous research has found relationships between specific indoor environmental exposures and exacerbation of asthma.

Objectives: In this review we provide an updated summary of knowledge from the scientific literature on indoor exposures and exacerbation of asthma.

Methods: Peer-reviewed articles published from 2000 to 2013 on indoor exposures and exacerbation of asthma were identified through PubMed, from reference lists, and from authors’ files. Articles that focused on modifiable indoor exposures in relation to frequency or severity of exacerbation of asthma were selected for review. Research findings were reviewed and summarized with consideration of the strength of the evidence.

Results: Sixty-nine eligible articles were included. Major changed conclusions include a causal relationship with exacerbation for indoor dampness or dampness-related agents (in children); associations with exacerbation for dampness or dampness-related agents (in adults), endotoxin, and environmental tobacco smoke (in preschool children); and limited or suggestive evidence for association with exacerbation for indoor culturable *Penicillium* or total fungi, nitrogen dioxide, rodents (nonoccupational), feather/down pillows (protective relative to synthetic bedding), and (regardless of specific sensitization) dust mite, cockroach, dog, and dampness-related agents.

Discussion: This review, incorporating evidence reported since 2000, increases the strength of evidence linking many indoor factors to the exacerbation of asthma. Conclusions should be considered provisional until all available evidence is examined more thoroughly.

Conclusion: Multiple indoor exposures, especially dampness-related agents, merit increased attention to prevent exacerbation of asthma, possibly even in nonsensitized individuals. Additional research to establish causality and evaluate interventions is needed for these and other indoor exposures.

Citation: Kanchongkittiphon W, Mendell MJ, Gaffin JM, Wang G, Phipatanakul W. 2015. Indoor environmental exposures and exacerbation of asthma: an update to the 2000 review by the Institute of Medicine. Environ Health Perspect 123:6–20; http://dx.doi.org/10.1289/ehp.1307922

## Introduction

Various allergens, airborne irritants, and infections trigger exacerbation of asthma. Because people spend > 75% of their time indoors (Klepeis et al. 2001), exposures found in the indoor environment are paramount. In 2000, the Committee on the Assessment of Asthma and Indoor Air of the Institute of Medicine (IOM) reviewed and summarized the scientific evidence for relationships between indoor air exposures and the exacerbation and development of asthma (IOM 2000). For exacerbation of asthma, sufficient evidence showed a causal relationship for four exposures and association for five exposures, and limited or suggestive evidence showed association with nine exposures. Since 2000, a substantial amount of research on indoor environmental exposures and exacerbation of asthma has been conducted. In this review we aim to provide a comprehensive update on this topic.

## Methods

We examined publications since 2000 relating indoor exposures and exacerbation of asthma in conjunction with prior evidence (IOM 2000) and provide conclusions about the current strength of evidence. We used a set of previously defined categories for strength of evidence (IOM 2000): *a*) sufficient evidence of causal relationship, *b*) sufficient evidence of an association, *c*) limited or suggestive evidence of an association, *d*) inadequate or insufficient evidence to determine whether or not an association exists, and *e*) limited or suggestive evidence of no association (for category descriptions, see Supplemental Material, Table S1).

### Specific Priorities, Inclusions, and Exclusions

In this review we considered the evidence that specific indoor environmental exposures might cause exacerbation of asthma. Eligible outcome measures, all among asthmatics, included frequency or severity of respiratory symptoms, illness-related school absences, urgent care or emergency department visits, hospitalization, unscheduled health care visits, amount or frequency of medicine for asthma control or prevention, airway inflammation assessed by fraction of exhaled nitric oxide (FeNO), and asthma-related quality of life.

Potentially modifiable biological and chemical exposures resulting from indoor sources were considered for inclusion as potential causes of asthma morbidity. Infectious agents and outdoor-generated pollutants that penetrate buildings were excluded. Studies on new onset of asthma, asthma prevalence, or experimental biologic markers of asthma were excluded.

Only studies of human health effects were included. Eligible study designs were controlled (experimental) exposure studies, environmental intervention studies, and a variety of observational designs: prospective or retrospective (longitudinal) cohort, case–control, and cross-sectional. Case studies and case series were ineligible. Detailed inclusion and exclusion criteria are described in the Supplemental Material, “Study inclusion criteria.”

### Literature Search

PubMed searches were performed in May and August 2011 and updated in August 2013. Search terms focused primarily on the indoor environmental risk factors considered in the IOM 2000 review. We added the category tag “major” to identify articles that included the IOM risk factor as a main topic and the broader category tag “mesh” to identify articles that included the IOM risk factors as a subject, but not necessarily as a main topic. This search strategy was designed to exclude editorials, letters, commentaries, clinical trials (phases 1–4) that would assess drug development and efficacy, and studies focusing on genetic predisposition or polymorphisms associated with asthma development. In addition, this search was restricted to findings published in English during the past 13 years. For further details regarding the search strategy, see Supplemental Material, “Literature search strategy.”

In total, the searches yielded 2,570 articles. After application of inclusion and exclusion criteria to the abstracts, we identified 162 articles of preliminary interest. We further excluded 99 studies after reviewing the full articles. Six additional peer-reviewed articles from reference lists or researchers’ files were included. Finally, 69 articles were selected for this review article. We considered recent findings in conjunction with evidence cited in the IOM (2000).

## Results

We organized evidence and conclusions by specific risk factors or exposures, ordered in categories by the previous strength of evidence for causation or association with exacerbation of asthma, as presented in the 2000 IOM review. Each section addresses a specific risk factor or exposure, presenting background material on the agent with conclusions from the IOM (2000) report, a summary of new evidence with consideration of prior evidence, and updated conclusions. Results are summarized in Table 1. A reevaluation of prior evidence cited in the IOM (2000) review is provided in Supplemental Material, “Prior evidence for selected exposures (IOM 2000).” Eligible studies included in the current review are listed in Supplemental Material, Tables S2–S13.

**Table 1:** Prior and updated conclusions about strength of evidence linking specific indoor exposures to increased exacerbation of asthma in asthmatic individuals.

Exposures according to prior strength of evidence, per IOM (2000)	Prior conclusions (IOM 2000)	Updated conclusions
Sufficient evidence for causation
House dust mite allergens	There is sufficient evidence of a causal relationship between dust mite allergen exposure and exacerbations of asthma in individuals specifically sensitized to dust mites. Continual exposure to dust mite allergens is also a contributing cause of chronic bronchial hyperreactivity.	There is sufficient evidence of a causal relationship between exposure to dust mite allergen and exacerbation of asthma in children sensitized to dust mites. There is limited or suggestive evidence of an association between dust mite allergen exposure and exacerbations of asthma in children not sensitized to dust mites and in adults, specifically sensitized or nonsensitized.
Cat allergens	There is sufficient evidence of a causal relationship between cat allergen exposure and exacerbation of asthma in individuals specifically sensitized to cats.	There is sufficient evidence of a causal relationship between cat allergen exposure and exacerbation of asthma in individuals specifically sensitized to cats.
Cockroach allergens	There is sufficient evidence of a causal relationship between cockroach allergen exposure and exacerbation of asthma in individuals specifically sensitized to cockroaches.	There is sufficient evidence of a causal relationship between cockroach allergen exposure and exacerbations of asthma in individuals specifically sensitized to cockroaches, especially adults. There is limited or suggestive evidence of association between cockroach allergen exposure and exacerbation of asthma in children not sensitized to cockroaches.
ETS	There is sufficient evidence to conclude that there is a causal relationship between chronic ETS exposure and exacerbations of asthma in preschool-age children. There is limited or suggestive evidence of a relationship between chronic ETS exposure and exacerbations of asthma in older children and adults. There is limited or suggestive evidence of an association between acute ETS exposure and exacerbation in asthmatics sensitive to this exposure.	There is sufficient evidence of an association between chronic ETS exposure and exacerbations of asthma in preschool-age children. There is limited or suggestive evidence of an association between chronic ETS exposure and exacerbations of asthma in older children and adults. There is limited or suggestive evidence of an association between acute ETS exposure and exacerbation of asthma in asthmatics sensitive to this exposure.
Sufficient evidence for association
Dog allergens	There is sufficient evidence of an association between dog allergen exposure and exacerbation of asthma in individuals specifically sensitized to dogs.	There is sufficient evidence of an association between dog allergen exposure and exacerbations of asthma in children sensitized to dogs. There is limited or suggestive evidence of an association between dog allergen exposure and exacerbations of asthma in nonsensitized adults.
Fungi (quantified)	There is sufficient evidence of an association between fungal exposure and symptom exacerbation in sensitized asthmatics. Exposure may also be related to nonspecific chest symptoms.	There is sufficient evidence of a causal association between outdoor culturable fungal exposure and exacerbation in asthmatics sensitized to fungi. There is limited or suggestive evidence of an association between indoor culturable *Penicillium* exposure and exacerbation in asthmatic children with specific sensitization, any fungal sensitization, or unspecified sensitization. There is limited or suggestive evidence of an association between indoor total culturable fungal exposure and exacerbation of asthma in children with any fungal sensitization.
Dampness or dampness-related agents	Dampness may exacerbate existing asthma. The factors related to dampness that actually lead to the development of disease and to disease exacerbation are not yet confirmed, but probably relate to dust mite and fungal allergens.	There is sufficient evidence of a causal association between dampness or dampness-related agents and exacerbation of asthma in children, and of an association in adults. The evidence does not suggest that this relationship is restricted to those with specific sensitization to fungi or dust mites.
Nitrogen dioxide (NO_2_)	There is sufficient evidence of an association between brief high-level exposures to NO_2_ and increased airway responses to both nonspecific chemical irritants and inhaled allergens among asthmatic subjects. These effects have been observed in human chamber studies at concentrations that may occur only in poorly ventilated kitchens with gas appliances in use.	There is sufficient evidence of an association between brief high-level exposures to NO_2_ and increased airway responses to nonspecific chemical irritants and inhaled allergens among asthmatic subjects. There is limited or suggestive evidence of an association between NO_2_ and exacerbation of asthma, although this association may be attributable to confounding by other consistently correlated emissions from gas stoves. There is inadequate or insufficient evidence to determine whether an association exists between gas stove use and exacerbation of asthma.
Limited or suggestive evidence for association
Domestic birds	There is limited or suggestive evidence of an association between bird exposure and exacerbation of symptoms in bird-sensitized asthmatics. This association may be confounded by the allergic asthmatic response to mites harbored by birds.	There is limited or suggestive evidence of an association between exposure to birds and the exacerbation of symptoms in bird-sensitized asthmatics. This association may be confounded by the allergic asthmatic response to mites harbored by birds.
Formaldehyde (nonoccupational)	There is limited or suggestive evidence of an association between formaldehyde exposure and wheezing and other respiratory symptoms.	There is limited or suggestive evidence of an association between formaldehyde exposure and exacerbations of asthma, particularly through enhanced response to other allergens.
Fragrances, some	There is limited or suggestive evidence of an association between exposure to certain fragrances and the manifestation of respiratory symptoms in asthmatics sensitive to such exposures.	There is limited or suggestive evidence of an association between exposure to certain fragrances and the manifestation of respiratory symptoms in asthmatics sensitive to such exposures.
Inadequate or insufficient evidence for association
Rodents (acute, nonoccupational)	There is sufficient evidence of an association between exposure to rodents in a laboratory setting and exacerbation of symptoms or lung function in rodent-sensitized asthmatics. There is inadequate or insufficient evidence to determine whether or not an association exists between exposure to rodents (wild or as pets) in the home and exacerbation of symptoms or lung function in rodent-sensitized asthmatics.	There is sufficient evidence of a causal association between exposure to rodents in a laboratory setting and exacerbation of symptoms or lung function in rodent-sensitized asthmatics. There is limited or suggestive evidence of an association between exposure to rats or mice in the home and increased exacerbation or severity of asthma in rodent-sensitized asthmatic children.
Cow and horse allergens (acute, nonoccupational)	There is inadequate or insufficient evidence to determine whether or not an association exists between cow or horse allergen in the home and the exacerbation of asthma in sensitive children.	There is inadequate or insufficient evidence to determine whether or not an association exists between cow or horse allergen in the home and the exacerbation of asthma in sensitive children.
Endotoxins (low level)	There is inadequate or insufficient information to determine whether or not an association exists between low-level indoor endotoxin exposure and exacerbation of asthma.	There is sufficient evidence of an association between indoor endotoxin exposure and the exacerbation of asthma.
Houseplants (+ cut flowers)	There is inadequate or insufficient evidence to determine whether or not an association exists between exposures from houseplants and the exacerbation of asthma.	There is inadequate or insufficient evidence to determine whether or not an association exists between exposures from houseplants and the exacerbation of asthma.
Pesticides (residential, nonoccupational)	There is inadequate or insufficient evidence to determine whether or not an association exists between pesticide exposures at the levels typically encountered in non-occupational or residential settings and the exacerbation of asthma.	There is inadequate or insufficient evidence to determine whether or not an association exists between pesticide exposures at the levels typically encountered in nonoccupational or residential settings and the exacerbation of asthma.
Plasticizers (nonoccupational)	There is inadequate or insufficient evidence to determine whether or not an association exists between nonoccupational exposure to plasticizers and the exacerbation of asthma.	There is inadequate or insufficient evidence to determine whether or not an association exists between nonoccupational exposure to plasticizers and the exacerbation of asthma.
Volatile organic compounds (VOCs) (residential, other than formaldehyde)	There is inadequate or insufficient evidence to determine whether or not an association exists between indoor residential VOC exposures and the exacerbation of asthma.	There is inadequate or insufficient evidence to determine whether or not an association exists between indoor residential VOC exposures (other than formaldehyde) and the exacerbation of asthma.
Pollen indoors	There is inadequate or insufficient evidence to determine whether or not an association exists between pollen exposure in the indoor environment and the exacerbation of asthma.	There is inadequate or insufficient evidence to determine whether or not an association exists between pollen exposure in the indoor environment and the exacerbation of asthma.
Down/feather bedding or synthetic bedding	There is inadequate or insufficient evidence to determine whether or not an association exists between down pillows and exacerbation of symptoms or lung function in asthmatics. Down pillows are believed to be a risk factor for asthma because of their documented mite content, rather than because of the presence of bird allergen.	There is limited or suggestive evidence that down/feather bedding may be protective for various respiratory effects relative to synthetic bedding, presumably due to lower content of dust mites, although evidence is not available regarding exacerbation of asthma.
Low outdoor air ventilation rate	There is inadequate or insufficient information to determine whether an association exists between lower ventilation rates and the exacerbation of asthma symptoms.	There is inadequate or insufficient evidence to determine whether an association exists between lower ventilation rates in buildings and the exacerbation of asthma.
ETS, environmental tobacco smoke. Exposures excluded from this review included all infectious agents (e.g., *Chlamydia pneumoniae, Chlamydia trachomatis, Mycoplasma pneumoniae,* respiratory syncytial virus) and all outdoor-generated pollutants (e.g., ozone, sulfur dioxide, nitrogen dioxide, nonbiologic particles).		

### Exposures for which Sufficient Evidence Had Existed for Causation of Exacerbation of Asthma

*House dust mite allergens*. Background. *Der p* 1 and *Der p* 2, the major allergens identified from house dust mites (*Dermatophagoides pteronyssinus*), are concentrated in particles of mite feces. Because the allergens are found on large particles (mostly ≥ 10 μm), they become airborne relatively briefly when disturbed. Studies have found that exposure to dust mite allergens is associated with dust mite sensitization, which is associated with asthma. Reduction of exposure to these allergens decreases asthma symptoms in mite-sensitive asthmatics (IOM 2000). Mechanistic studies have implicated both allergic and nonallergic mechanisms in inducing airway inflammation, mirroring some clinical findings of dust mite–induced asthma morbidity in nonsensitized subjects. Protease activity of dust mite allergen can act on airway epithelial cells to induce disruption of the tight junctions between epithelial cells and activation of protease-activated receptor-2 (PAR-2), facilitating allergen delivery across epithelium (Jeong et al. 2008). Activation of PAR-2 can trigger an innate immune response and the release of proinflammatory cytokines, such as interleukin-6 (IL-6) and IL-8 from airway epithelial cells (Kauffman 2006; Matsumura 2012). Citing the many studies that had found exposure to dust mite allergens to be associated with dust mite sensitization—which was in turn associated with asthma—and reduction of exposure to dust mite allergens to be associated with decreased asthma symptoms in mite-sensitive asthmatics, the IOM concluded:

- There is sufficient evidence of a causal relationship between dust mite allergen exposure and exacerbations of asthma in individuals specifically sensitized to dust mites. Continual exposure to dust mite allergens is also a contributing cause of chronic bronchial hyperreactivity (IOM 2000).

Summary of new evidence. In the Supplemental Material, Table S2 summarizes recent reported findings from 11 studies on associations of dust mite allergens with exacerbation of asthma: in children, one randomized controlled trial, four prospective studies, and four cross-sectional studies (including one with case–control selection but cross-sectional analysis); in adults, one randomized control trial; and in children and adults, one cross-sectional study with findings reported only for adults.

In specifically sensitized children, the intervention trial (El-Ghitany and Abd El-Salam 2012) and all four prospective studies (Gent et al. 2009, 2012; Halken et al. 2003; Nitschke et al. 2006) found associations of dust mite exposure with exacerbation, with Gent et al. (2009) reporting a dose-related association of dust mite antigen with wheeze. Of three cross-sectional assessments, one (Spanier et al. 2006) found significant positive associations of dust mite exposure with exacerbation, one (Murray et al. 2006) found a nonsignificant increase, and one (Rabito et al. 2011) found no association. In children not specifically sensitized, the only prospective study (Gent et al. 2009) and one (Spanier et al. 2006) of two (Rabito et al. 2011) cross-sectional studies found associations. In children of unknown sensitization, the one study (Turyk et al. 2006) found no association. In adults, the one study in specifically sensitized adults found no greater benefits associated with a dust mite reduction intervention than with control conditions (Dharmage et al. 2006). In adults who were not specifically sensitized but were atopic asthmatics, a cross-sectional study found that exposure to dust mite allergen was significantly associated with more severe bronchial hyperresponsiveness (Langley et al. 2005). Spanier et al. (2006) reported a positive association between dust mite allergen level and FeNO in dust mite–sensitized children, suggesting a direct role in airway inflammation.

Evidence from the IOM (2000), while clearly documenting that dust mite allergen exposure caused asthma in dust mite–sensitive children, was less clear in documenting that such exposure caused exacerbation of existing asthma. The strongest evidence for this—the available intervention studies—also involved many changes other than dust mite reduction. In the evidence currently available, dust mite exposure consistently is positively associated with various measures of exacerbation or severity of asthma in children, including in studies of strong design, with proper temporality, and with biologic plausibility demonstrated. In children not specifically sensitized the evidence is more sparse, although more positive than negative. In adults the sparse evidence is not consistent, but only suggestive.

### Conclusions.

- There is sufficient evidence of a causal relationship between exposure to dust mite allergen and exacerbation of asthma in children sensitized to dust mites.
- There is limited or suggestive evidence of an association between dust mite allergen exposure and exacerbations of asthma in children not sensitized to dust mites and in adults, whether sensitized or nonsensitized.

*Cat allergen*. Background. *Fel d* 1 has been identified as the major cat allergen. High levels of *Fel d* 1 are found in the air and dust of homes with cats, but are also found in many buildings without resident cats (IOM 2000). The mix of prior findings in studies of cat allergens, atopy, and asthma was complex, possibly suggesting different effects depending on age at exposure, level of exposure, and sensitization status. Early exposure to cats appeared possibly to prevent sensitization to cat allergens in some children, although findings were inconsistent. The IOM concluded:

- There is sufficient evidence of a causal relationship between cat allergen exposure and exacerbation of asthma in individuals specifically sensitized to cats (IOM 2000).

Summary of new evidence. In the Supplemental Material, Table S3 summarizes evidence from seven recent available studies: in children, three prospective and three cross-sectional, and in adults and children, one cross-sectional.

The prospective study by McConnell et al. (2006) found that associations of various air pollutants with bronchitic symptoms were generally greater among children living in a home with a cat. Several studies have also demonstrated that among cat-sensitized children, cat allergen exposure had a positive association with increased asthma severity, rescue medication use, frequency of asthma symptoms, or FeNO: two prospective studies (Gent et al. 2009, 2012) and one cross-sectional (Murray et al. 2006). In other cross-sectional studies, Turyk et al. (2006) found no significant associations, and Spanier et al. (2006) found that high levels of cat allergen exposure and owning a cat were both associated with lower FeNO (i.e., less airway inflammation). This last finding may be biased by cat-allergic subjects tending not to own cats. In asthmatic adults who were atopic but not sensitized to cat, no association was observed between *Fel d* 1 and asthma, including measures of forced expiratory volume in 1 sec (FEV_1_), provocation dose causing fall in FEV_1_ of at least 20% (PD20), and FeNO (Langley et al. 2005).

Thus, findings in children from prospective studies are fully consistent, and those from cross-sectional studies partly consistent, with prior findings that cat allergen exposure among cat-sensitized individuals (presumably with inflamed airways) increases asthma symptoms.

### Conclusion.

- There is sufficient evidence of a causal relationship between cat allergen exposure and exacerbation of asthma in individuals sensitized to cats.

*Cockroach allergens*. Background. The major cockroach allergens include enzymes in fecal pellets that are incorporated into household dust (Rabito et al. 2011). Cockroach allergen exposure is common in inner-city residences and was considered to be an important influence on the high rates of asthma among inner-city children (IOM 2000). The evidence cited by the IOM (2000) was limited but consistent, with two positive controlled exposure studies showing responses in specifically sensitized adults and one positive prospective observational study in children. There was documented association, biologic plausibility, and apparent causality in specifically sensitized individuals. The IOM concluded:

- There is sufficient evidence of a causal relationship between cockroach allergen exposure and exacerbation of asthma in individuals specifically sensitized to cockroaches (IOM 2000).

Summary of new evidence. In the Supplemental Material, Table S4 summarizes findings from seven recent studies: one prospective and six cross-sectional, all in children; one study also included adults.

No new controlled exposure studies were identified, but those cited by the IOM (2000) provided strong evidence confirming causality in sensitized adults (Bernton et al. 1972; Kang 1976). In children, controlled exposure studies have not been available. Prospective findings in children have been uniformly positive. For prospective studies, the one recent and one prior study, in specifically sensitized children, both found associations of bedroom cockroach allergen with increased asthma morbidity: allergen in bedroom floor and bed dust with significantly more asthma symptom days, school days missed, and caretaker-interrupted sleep in Gruchalla et al. (2005), and allergen in bedroom floor dust with more than three times the rate of hospitalization in Rosenstreich et al. (1997).

In contrast, cross-sectional findings in children on bedroom cockroach allergen, all recent, have been mixed. Gent et al. (2009) found no associations of cockroach allergen in beds with asthma severity in children with, or those without, specific sensitization. This study, however, had two limitations: Allergen levels were very low, with only 4% of children exposed at > 2 μg/g of dust, the level with increased risk in Gruchalla et al. (2005), compared with 35% in that study, and dust samples were taken only from beds, whereas all positive studies included bedroom floor dust. In children of unknown atopic status, Turyk et al. (2006) found strong, dose-related associations of cockroach allergen in bedroom floor and bed dust with number of symptoms, with odds ratios (ORs) up to 5.8, but Spanier et al. (2006) found no associations of allergen in bedroom floor dust with FeNO.

For cross-sectional findings on cockroach allergen measured in kitchens, Rabito et al. (2011) reported strong associations (ORs > 4) with hospital admissions in both those with and those without specific sensitization. In cross-sectional findings based on other exposure assessments, neither of the two reports on measured cockroach antigens in living rooms found associations with exacerbation of asthma (Gent et al. 2009; Turyk et al. 2006); and neither of the two studies on visible cockroach presence in homes found associations with exacerbation of asthma: with asthma symptoms, unscheduled medical visits, or steroid use for asthma attacks (Bonner et al. 2006), and with asthma symptoms (Shedd et al. 2007).

The strong association of cockroach allergen with severe effects in both sensitized and nonsensitized children in one study (Rabito et al. 2011) might be attributable to irritant effects or non-IgE–mediated sensitization mechanisms. Cockroach allergen can induce mucosal allergic sensitization and inflammation via PAR-2 (Jeong et al. 2008), induce expression of inflammatory cytokine (e.g., IL-8, IL-6), and trigger innate immune response in the human airway epithelium (Kauffman 2006; Matsumura 2012). These findings suggest a potential mechanism for cockroach allergen-induced asthma morbidity in sensitized and nonsensitized subjects.

The overall evidence demonstrates that exposure to cockroach antigen causes exacerbation of asthma in specifically sensitized adults, but such evidence is less consistent for children. For specifically sensitized children, the strongest studies, the two prospective, consistently show associations with measured allergen exposures in bedrooms, which may be a critical location for exposure. Although cross-sectional findings for bedroom dust are mixed, the only one with negative findings for sensitized children had very low exposure levels. Other cross-sectional study findings are limited but positive for kitchen exposures, and negative for exposures in living rooms or as assessed by visible cockroaches in homes. A few reported associations are strong (Rabito et al. 2011; Rosenstreich et al. 1997; Turyk et al. 2006), including a strong finding in nonsensitized children, and dose–response findings are available from one study (Turyk et al. 2006). The most reasonable conclusion is still of a causal relationship in both adults and children, although the evidence in children is weaker. Strong study designs and improved exposure assessment may be key to future demonstration of health risks associated with cockroach allergen.

### Conclusions.

- There is sufficient evidence of a causal relationship between cockroach allergen exposure and exacerbation of asthma in individuals specifically sensitized to cockroaches, especially adults.
- There is limited or suggestive evidence of association between cockroach allergen exposure and exacerbation of asthma in children not sensitized to cockroaches.

*Environmental tobacco smoke exposures*. Background. A substantial body of research has assessed the respiratory health effects of involuntary exposure to environmental tobacco smoke (ETS). Tobacco smoke contains solid particles and semivolatile and volatile organic compounds. These compounds include known or suspected eye and respiratory irritants, toxicants, mutagens, and carcinogens (Zeise and Dunn 1999). Distinguishing the effects of acute versus chronic ETS exposure is challenging, except by controlled chamber studies, which have been conducted only in adults (IOM 2000). Therefore, all observational studies are considered to assess chronic ETS exposure. The IOM concluded:

- There is sufficient evidence of a causal relationship between chronic ETS exposure and exacerbations of asthma in preschool-age children.
- There is limited or suggestive evidence of a relationship between chronic ETS exposure and exacerbations of asthma in older children and adults.
- There is limited or suggestive evidence of an association between acute ETS exposure and exacerbation in asthmatics sensitive to this exposure (IOM 2000).

Summary of new evidence. In the Supplemental Material, Table S5 summarizes 19 recent studies on ETS exposure and exacerbation of asthma: in preschool-age children, two prospective studies; in older children, three prospective, one case–control, and eight cross-sectional studies; in adults and children, one prospective and one cross-sectional study, and in adults, three prospective studies. Some new findings have failed to show a relationship between ETS and exacerbation of asthma.

In preschool-age children, Kattan et al. (2007) found that high ETS exposures were not significantly related to increased wheeze or unscheduled medical visits, though they were associated with decreased peak flow in cold weather. Perzanowski et al. (2010) reported that, although previous ETS exposure was positively associated with FeNO, current exposure was inversely related to FeNO. ETS exposure at 4 years of age was associated with significantly lower FEV_1_ and forced expiratory flow in mid 50% of exhaled volume (FEF_25–75%_) at 7 years, but not concurrent exposure at 7 years.

In older children with asthma, FeNO was not associated with reported tobacco smoke exposure, serum cotinine, or hair cotinine (Dinakar et al. 2005; Spanier et al. 2006, 2008). In fact, measured nicotine exposure was associated with decreased FeNO (Spanier et al. 2008), a response possibly mediated by a *NOS3* genetic polymorphism (Spanier et al. 2009). Glutathione *S*-transferase gene variants have also been implicated in lung function response to ETS (Palmer et al. 2006). This may indicate that ETS does not cause allergic airway inflammation. Other recent studies demonstrate no difference in acute asthma symptoms or responses to emergency therapy by ETS exposure (Karadag et al. 2003; Vargas et al. 2007).

In contrast, many studies in children have continued to demonstrate associations of ETS with reduced lung function, increased wheezing, nocturnal symptoms, and emergency department visits (Chapman et al. 2003; Lawson et al. 2011; Morkjaroenpong et al. 2002; Soussan et al. 2003; Sturm et al. 2004; Wang et al. 2007). Ecologic evidence presented by Herman and Walsh (2011) on adults and children demonstrated 22% lower hospital admission rates for asthma after a smoking ban in Arizona, compared with rates in counties without bans. In adults, Eisner et al. (2002, 2005) demonstrated that ETS exposure was associated with greater asthma severity during follow-up, emergency department visits, and hospital admissions for asthma. Newman et al. (2010) reported that home ETS exposure had a nonsignificant association with reduced severity or frequency of symptoms during pregnancy.

Overall, regarding ETS and exacerbation of asthma in preschool-age children, the basis for the prior IOM conclusion of demonstrated causality is not clear. The two recent prospective studies in preschool-age children (Kattan et al. 2007; Perzanowski et al. 2010) have not found the clear relationships expected from a causal relationship. The overall evidence now, not fully consistent, seems to better support a finding of an association rather than of causality for preschool-age children. For older children and adults, available evidence is very inconsistent regarding association of ETS with increased asthma morbidity. This is in agreement with the prior assessment of limited or suggestive evidence of a relationship. No additional recent evidence was identified regarding acute ETS exposure and exacerbation of asthma.

### Conclusions.

- There is sufficient evidence of an association between chronic ETS exposure and exacerbations of asthma in preschool-age children.
- There is limited or suggestive evidence of an association between chronic ETS exposure and exacerbations of asthma in older children and adults.
- There is limited or suggestive evidence of an association between acute ETS exposure and exacerbation of asthma in asthmatics sensitive to this exposure.

### Exposures for which Sufficient Evidence Had Existed for an Association with Exacerbation of Asthma

*Dog allergens*. Background. Two major dog allergens, *Can f* 1 and *Can f* 2, have been identified. High levels of dog dander have been found in homes with dogs and in homes and buildings without resident dogs, and positive bronchial provocation tests with dog allergen have been correlated with sensitization to dog allergen (IOM 2000). The IOM concluded:

- There is sufficient evidence of an association between dog allergen exposure and exacerbation of asthma in individuals specifically sensitized to dog allergen (IOM 2000).

Summary of new evidence. In the Supplemental Material, Table S6 summarizes the findings of five recent available studies: in children, three prospective and one cross-sectional, and in adults, one cross-sectional.

McConnell et al. (2006) found that the presence of dogs in homes increased the effect of ambient pollutants on bronchitic symptoms in asthmatic children not assessed for specific sensitization. Two other prospective studies and the cross-sectional study found that exposure to dog allergens significantly increased asthma severity in sensitized children (Gent et al. 2009, 2012; Murray et al. 2006). In adults, nonsensitized subjects exposed to high levels of dog allergens also demonstrated increased airway reactivity (Langley et al. 2005).

Prior evidence was sufficient only to suggest but not (as the IOM concluded) to establish associations between indoor dog allergen exposure and exacerbation of asthma. The addition of current findings establishes an association of dog allergen exposure to exacerbation of asthma in sensitized children, and also suggests associations in nonsensitized adults.

### Conclusions.

- There is sufficient evidence of an association between dog allergen exposure and exacerbations of asthma in children sensitized to dogs.
- There is limited or suggestive evidence of an association between dog allergen exposure and exacerbations of asthma in nonsensitized adults.

*Fungi (quantified)*. Background. In this section we review studies of quantified fungal exposures as triggers of asthma. Effects of qualitatively assessed environmental fungi or of fungal components, fungal by-products, or other dampness-related agents are reviewed in the next section, “Dampness or dampness-related agents.”

Sensitization to many fungal species that occur outdoors and indoors has long been documented, but the mechanisms underlying the responses to most fungi have not been fully defined. Increased asthma severity with higher outdoor fungal spore concentrations, provocation of asthma symptoms among patients with fungal sensitivities, and reduction in asthma symptoms by desensitization with fungal antigens have been demonstrated repeatedly (IOM 2000). However, links between measured indoor fungal concentrations and exacerbation of asthma, including relevant exposure parameters and mechanisms, have been less clear. The IOM concluded:

- There is sufficient evidence of an association between fungal exposure and symptom exacerbation in sensitized asthmatics. Exposure may also be related to nonspecific chest symptoms (IOM 2000).

Summary of new evidence. The studies previously cited by the IOM (2000), in conjunction with Atkinson et al. (2006) and Pongracic et al. (2010), demonstrate that outdoor fungal exposures cause exacerbation of asthma in sensitized individuals. Atkinson et al. (2006), for instance, reported that outdoor fungal concentrations were positively related to children’s admissions to emergency rooms, after adjustment for pollen and air pollutants. The overall evidence documents strong association, temporality, consistency, and biologic plausibility, with dose-related response shown by Pongracic et al. (2010).

In the Supplemental Material, Table S7 summarizes six studies providing recent evidence on indoor fungal exposure and exacerbation of asthma, all in children: five prospective and one cross-sectional. All findings described below, unless otherwise specified, refer to indoor culturable airborne fungal concentrations, measured in prospective studies in asthmatic children, with analyses adjusted for key confounding variables.

Two prospective studies found that in specifically sensitized children, culturable airborne indoor *Penicillium* was associated with significantly increased outcomes of severe exacerbations (as indicated by unscheduled medical visits), severity, or symptoms (Gent et al. 2012; Pongracic et al. 2010), even after adjustment for outdoor fungal levels in one study (Gent et al. 2012). Among children with any fungal sensitization but not necessarily to the genus in question (26% were sensitized to *Penicillium*), Pongracic et al. (2010) found indoor culturable airborne *Penicillium*, and also the summed four most common fungal genera, to be associated with significantly increased severe exacerbations and symptoms, and total indoor fungal concentration to be associated with significantly increased severe exacerbations. Among those with any fungal sensitization, increases in symptom days associated with each specific genus were smaller among those not specifically sensitized than among those sensitized, although the decrease for *Penicillium* was only 5% (Pongracic et al. 2010).

Among asthmatic children of unknown sensitization status, a cross-sectional study by Turyk et al. (2006) found indoor *Penicillium* to be associated with significantly increased symptom frequency, and a prospective study by Bundy et al. (2009) found indoor *Penicillium,* but not total indoor fungi, to be associated with significantly increased peak expiratory flow variability (PEFV). In contrast, in unadjusted analyses from a small study of 19 children, Inal et al. (2007) found no significant associations between total indoor molds or four specific genera including *Penicillium,* and symptom or lung function outcomes; limitations of this study reduce its importance. An important weakness of these studies on airborne culturable fungi is that all those specifying their sampling methods used 1-min air samples, and thus had highly unreliable estimates of fungal concentrations, which have high temporal variability. In unadjusted analyses in children of unspecified atopic status, Wu et al. (2010) found that total culturable fungi in dust were associated with an increased number of urgent care visits only in those with genetic polymorphisms that caused reduced enzymatic breakdown of chitin, an important fungal protein. (This study provides biologic plausibility for nonallergic mechanisms for exacerbation of asthma in relation to fungal exposures.)

The overall available evidence is sufficient to document that asthma exacerbation is caused by outdoor fungal exposures in those sensitized. Although these outdoor fungi also occur indoors, the evidence is not sufficient to demonstrate causality or association directly from indoor fungal exposures. The associations reported for *Penicillium* may be attributable to confounding by other indoor dampness-related exposures.

### Conclusions.

- There is sufficient evidence of a causal association between outdoor culturable fungal exposure and exacerbation in asthmatics sensitized to fungi.
- There is limited or suggestive evidence of an association between indoor culturable *Penicillium* exposure and exacerbation in asthmatic children with specific sensitization, any fungal sensitization, or unspecified sensitization.
- There is limited or suggestive evidence of an association between indoor total culturable fungal exposure and exacerbation of asthma in children with any fungal sensitization.

*Dampness or dampness-related agents*. Background. Sufficient evidence has long been available to document associations between indicators of dampness and exacerbation of asthma and other respiratory effects. The specific dampness-related causal agents, although not identified, were assumed to be dust mite or fungal allergens (IOM 2000). The few available studies showed fairly consistent, strong associations between dampness indicators in buildings and exacerbation of asthma. The prior IOM conclusion did not clearly specify if evidence demonstrated causality or only association:

- Dampness may exacerbate existing asthma. The factors related to dampness that actually lead to the development of disease and to disease exacerbation are not yet confirmed, but probably relate to dust mite and fungal allergens (IOM 2000).

Summary of new evidence. Eight studies on qualitative assessments of dampness indicators (e.g., visible dampness, water damage, or mold, or mold odor) or quantified dampness and exacerbation of asthma have become available or were not previously considered. In the Supplemental Material, Table S8 summarizes evidence from these eight studies: in children, two controlled intervention, two prospective, and two cross-sectional studies; and in adults, one prospective and one cross-sectional study.

Kercsmar et al. (2006), in a controlled intervention study in damp houses of asthmatic children, reported that comprehensive remediation of dampness sources and visible mold caused dramatic reductions in severe exacerbation of asthma. Acute care visits after remediation were significantly reduced (by 90%) in the children in homes actually receiving remediation (i.e., as treated) compared with those in the control homes. Another intervention study (Bernstein et al. 2006) and two prospective studies (Hagmolen of Ten Have et al. 2007; Venn et al. 2003) in children found significant positive associations between dampness or mold and exacerbation or severity of asthma outcomes. Bernstein et al. (2006) performed a controlled intervention in which ultraviolet radiation was applied in home ventilation ducts to reduce microbial exposures to fungally sensitized asthmatic children. Ultraviolet radiation was associated with a significant reduction in PEFV and a nonsignificant reduction in FEV_1_; with significant reductions in severity scores for shortness of breath and chest tightness, in number of days of shortness of breath and chest tightness, and in amount of medication used; and with nonsignificant reductions in all other disease severity measures (Bernstein et al. 2006). The presumed mechanism is reduction of unspecified microbial exposures. The intervention study by Burr et al. (2007) was considered ineligible because its intervention for mold removal also increased outdoor air ventilation, which reduces concentration of other indoor airborne contaminants, thus making the study benefits not specific to mold removal.

Venn et al. (2003) reported a dose-related positive association of measured wall moisture with wheezing in children with persistent wheezing over 3 years, significantly more in atopic cases. ORs for measured bedroom moisture and nighttime symptoms, and measured living room moisture and daytime symptoms, both showed dose-related responses, with ORs ranging up to 7.0 for the highest moisture level. Visible mold was not significantly associated with either symptom type, but was significantly associated with presence of wheezing illness. Hagmolen of Ten Have et al. (2007) reported that damp stains or mold growth were significantly positively associated with three asthma severity metrics. Only one-third of the subjects were fungally sensitized.

Of two cross-sectional studies in children, Bonner et al. (2006) found strong positive associations between home moisture or mold and three asthma severity metrics, but Teach et al. (2006) found no such relationships. Bonner et al. (2006) reported the presence of moisture or mildew at home to be associated with more than three times the hospital visits for breathing-related problems, more than three times the frequency of wheezing episodes, and more than twice the expected frequency of night symptoms. Teach et al. (2006) reported that visible dampness or mold in the home during the previous month was not associated with unscheduled medical care visits above the median, persistent asthma symptoms, or quality-of-life scores below the median.

In adults, a prospective study found a doubling in asthma attacks with home mold exposure (Wen et al. 2009), regardless of subject obesity. A cross-sectional study in diagnosed asthmatics, previously cited by IOM (2000), found that both moisture meter–measured total home dampness and visible mold score had significant positive dose-related associations with asthma severity, and higher measured dampness was associated with significantly greater measured airflow obstruction (Williamson et al. 1997).

The specific causal agents for exacerbations of asthma that are associated with dampness have not been identified. Although it is often assumed that these agents are fungal, they may include other biologic exposures such as bacteria, amoebas, or dust mites that thrive in dampness, or nonbiologic exposures such as chemicals emitted from damp materials (Mendell et al. 2011).

In these studies, evident dampness or mold or measured dampness was positively associated—with almost complete consistency—with exacerbation or severity of asthma. The intervention studies, which had the strongest designs, both showed these relationships clearly. The intervention and prospective studies, both demonstrating temporality of effects, were consistent, as were all but one of the cross-sectional studies. Both Venn et al. (2003) and Williamson et al. (1997) showed strong, dose-related positive associations between measured moisture and asthma severity outcomes. Because of the implausibility of noncausal explanations for all these findings, especially those of Kercsmar et al. (2006), this evidence indicates a causal association between indoor dampness or dampness-related agents and exacerbation of asthma in children with asthma. Few of the populations in these studies were restricted to atopic subjects, much less to those sensitized to any fungi, specific fungal genera, or dust mites.

### Conclusion.

- There is sufficient evidence of a causal association between dampness or dampness-related agents and exacerbation of asthma in children, and of an association in adults. The evidence does not suggest that this relationship is restricted to those with specific sensitization to fungi or dust mites.

*Nitrogen dioxide*. Background. Nitrogen dioxide (NO_2_) is a common pollutant gas found indoors and outdoors. It is produced, along with other oxides of nitrogen, whenever high-temperature combustion occurs. Indoor combustion sources, including gas stoves and space heaters, kerosene space heaters, and poorly vented furnaces and fireplaces, produce high indoor NO_2_ concentrations. Individual NO_2_ exposures in homes equipped with combustion appliances are usually driven by concentrations generated indoors even when elevated outdoor levels infiltrate into the home. Indoor NO_2_ levels in homes equipped with gas stoves are higher in kitchens than in other rooms and greatly higher during cooking. Thus, individual exposure in a home depends heavily on the amount of time spent in the kitchen during cooking (IOM 2000).

In addition to nitrogen oxides, indoor combustion appliances generally emit a variety of other pollutants such as carbon monoxide, sulfur dioxide (SO_2_), formaldehyde, volatile organic compounds (VOCs), and submicron particulate matter (PM), some of which are known respiratory irritants. Epidemiologic studies generally have used only the presence of gas appliances as an indicator of elevated indoor NO_2_, or have measured NO_2_ but not other gas combustion emissions. Such studies thus have not been able to attribute health effects associated with gas appliances, or even with measured NO_2_, to NO_2_ exposure itself (IOM 2000). Brief, controlled high-level NO_2_ exposures, such as might be found in poorly ventilated kitchens during gas appliance use, caused enhanced airway responsiveness in asthmatic adults (IOM 2000). The IOM concluded:

- There is sufficient evidence of an association between brief high-level exposures to NO_2_ and increased airway responses to both nonspecific chemical irritants and inhaled allergens among asthmatic subjects. These effects have been observed in human chamber studies at concentrations that may occur only in poorly ventilated kitchens with gas appliances in use (IOM 2000).

Summary of new evidence. In the Supplemental Material, Table S9 summarizes recent evidence from eight studies of NO_2_ and exacerbation of asthma: in children, two controlled intervention, four prospective cohort, and one cross-sectional study; and in adults, one prospective study. In the Supplemental Material, Table S10 summarizes the evidence from six studies of gas stove use and exacerbation of asthma: in children, three cross-sectional, and in adults, two prospective and one cross-sectional study.

For NO_2_ studies in children, the two intervention studies (Gillespie-Bennett et al. 2011; Pilotto et al. 2004) replaced unflued gas heaters that emitted NO_2_ and other combustion products indoors. Either unreplaced gas heaters or higher NO_2_ were associated with increased asthma symptoms (Gillespie-Bennett et al. 2011; Pilotto et al. 2004) and/or reduced FEV_1_ (Gillespie-Bennett et al. 2011). In prospective studies, higher indoor NO_2_ indoors was associated with increases in many but not all measures of asthma-related morbidity (Belanger et al. 2013; Fu et al. 2012; Hansel et al. 2008; Kattan et al. 2007). In the study by Belanger et al. (2013), four outcomes had significant dose-related increases. However, in one study, this association was found for wheeze only in nonatopic children (Kattan et al. 2007). Fu et al. (2012) found high NO_2_ exposures associated with more severe asthma in children with high beta-2-adrenergic receptor (*ADRB2*) gene methylation. In a cross-sectional study, Belanger et al. (2006) found that measured NO_2_ concentrations had a significant positive association with increased likelihood of wheeze and chest tightness among asthmatic children in multi-family housing, but not in single-family housing. In adults, a prospective study (Ng et al. 2001) found adverse respiratory effects in asthmatic women associated with cooking-related measured NO_2_ exposures.

Regarding gas stove use, two cross-sectional studies in children found associations with some but not all studied respiratory effects. Belanger et al. (2006) found that the presence of a gas stove in the home had a significant positive association with wheeze, shortness of breath, and chest tightness among children living in multi-family housing, but not single-family housing. Chapman et al. (2003) found that use of a gas stove in the home for cooking had a significant inverse relationship on FEF_25–75%_, FEF_25–75%_/forced vital capacity (FVC), FEV_1_, and FEV_1_/FVC among girls who did not take prescription respiratory medication. There was no association among girls when respiratory medication was taken, and no association at all among boys. Use of an exhaust fan had no influence on the effects of using a gas stove (Chapman et al. 2003). Bonner et al. (2006) found that the presence of a gas stove not equipped with an outside vented exhaust was not associated with any measure of asthma severity related to symptoms, missed school, unscheduled health care visits, or steroid-treated asthma attacks.

Studies of gas cooking in adults included two prospective cohort studies and one cross-sectional study. Ng et al. (2001), in a prospective study, found adverse respiratory effects in asthmatic women from both short-term and repeated cooking exposures. Eisner et al. (2002) reported from a prospective study that personal use of a gas stove for cooking was not associated with asthma severity score, use of systemic corticosteroids or other asthma medications, or a history of hospitalizations and intubations. In a cross-sectional study, Eisner and Blanc (2003) reported that gas stove use had no significant association with FEV_1_, FVC, FEV_1_/FVC ratio, or FEF_25–75%_ and no association with chronic cough or phlegm production. Gas stove use was related to a greater risk of dyspnea, wheeze, and any respiratory symptom, although those relationships were not statistically significant (Eisner and Blanc 2003).

The overall available evidence leaves unchanged, due to lack of new evidence, the IOM (2000) conclusion about brief high-level NO_2_ exposures in asthmatics. Substantial new evidence, although not fully consistent, shows associations between indoor NO_2_ exposure and exacerbation of asthma. Conclusions on this relationship are further limited by the fact that many other unmeasured combustion-related chemical and particulate compounds are emitted by gas stoves and heaters that may be related to asthma morbidity. Evidence for this relationship is thus considered to be only suggestive. Findings on gas stove use and exacerbation of asthma are too inconsistent to demonstrate associations.

### Conclusions.

- There is sufficient evidence of an association between brief high-level exposures to NO_2_ and increased airway responses to nonspecific chemical irritants and inhaled allergens among asthmatic subjects.
- There is limited or suggestive evidence of an association between NO_2_ and exacerbation of asthma, although this association may be attributable to confounding by other consistently correlated emissions from gas stoves.
- There is inadequate or insufficient evidence to determine whether an association exists between gas stove use and exacerbation of asthma.

### Exposures for which Limited or Suggestive Evidence had Existed for an Association with Exacerbation of Asthma

*Domestic birds*. Background. Respiratory allergies to birds have been known to occur among zoo keepers and pet shop workers, but specific links between exposures and exacerbations of asthma have not been made. The IOM review found that, although asthmatic symptoms had been documented in association with bird-keeping, specific bird antigens associated with allergies and asthma had not been identified and available evidence was lacking.

There was an assumption that feather bedding was associated with exacerbation of asthma because of mites associated with birds, but no supportive evidence was found. The IOM report cited only one epidemiologic study, which found increased risk of wheeze in children using foam rather than feather pillows, and theorized that this unexpected association might result from bias due to parental replacement of feather bedding by synthetic bedding for symptomatic children (Strachan and Carey 1995). The IOM review also cited findings of higher dust mite antigen levels in synthetic pillows than in feather pillows and theorized the difference might result from more impermeable covers on feather bedding preventing infestation (IOM 2000). The IOM review concluded

- There is limited or suggestive evidence of an association between bird exposure and exacerbation of symptoms in bird-sensitized asthmatics. This association may be confounded by the allergic asthmatic response to mites harbored by birds.
- There is inadequate or insufficient evidence to determine whether or not an association exists between down pillows and exacerbation of symptoms or lung function in asthmatics. Down pillows are believed to be a risk factor for asthma because of their documented mite content, rather than because of the presence of bird allergen (IOM 2000).

Summary of new evidence. No additional evidence was available on exposure to domestic birds and exacerbation of asthma over the past decade. Evidence continues to suggest that occupational exposure is associated with the development of antibodies to feathers from specific birds (Renström et al. 2011; Swiderska-Kielbik et al. 2011), but that allergic responses may also occur to mites harbored by birds (Rimac et al. 2010). Down/feather bedding is discussed as a separate exposure, below.

### Conclusion.

- There is limited or suggestive evidence of an association between exposure to birds and the exacerbation of symptoms in bird-sensitized asthmatics. This association may be confounded by the allergic asthmatic response to mites harbored by birds.

*Formaldehyde (nonoccupational)*. Background. Formaldehyde, an aldehyde and VOC, is emitted from many building materials, items of furniture, and consumer products, and by combustion processes including those in gas stoves and tobacco smoking. Cigarette smoke is considered to be one of the largest indoor sources of formaldehyde. The strongest sources have been urea–formaldehyde foam insulation (no longer used) and various composite wood products made with urea-formaldehyde resins. Formaldehyde is also present in outdoor air, where motor vehicle exhaust is a major source. Indoor concentrations are determined by the presence, number, and age of sources, modified by the rate of outdoor air ventilation. Formaldehyde concentrations are generally highest in newly constructed or renovated building spaces and in areas that contain new furnishings made with formaldehyde resins (IOM 2000).

High-level exposure to formaldehyde has been documented to cause occupational asthma, although whether this occurs through immunologic or irritant mechanisms has been unclear. Little evidence was available related to effects of residential exposures on exacerbation of asthma (IOM 2000). The IOM concluded:

- There is limited or suggestive evidence of an association between formaldehyde exposure and wheezing and other respiratory symptoms (IOM 2000).

Summary of new evidence. In the Supplemental Material, Table S11 summarizes the findings of two recent studies, both controlled exposure studies. In a blinded crossover study (Casset et al. 2006), exposure to 32 or 92 μg/m^3^ formaldehyde for 30 min had no effect on lung function or symptoms in asthmatic adults, but enhanced both immediate and late responses to dust mite antigen. Formaldehyde thus increased effects of a common asthma trigger without having apparent direct effects (Casset et al. 2006). A similar lack of lung function effect of 500 μg/m^3^ formaldehyde exposure for 60 min was demonstrated in patients with intermittent asthma (Ezratty et al. 2007). Although epidemiologic studies have shown associations of indoor formaldehyde exposures with asthma development and prevalent asthma in children (reviewed by McGwin et al. 2010), evidence on exacerbation of asthma was not available.

### Conclusion.

- There is limited or suggestive evidence of an association between formaldehyde exposure and exacerbation of asthma, particularly through enhanced response to other allergens.

*Exposure to certain fragrances (among sensitized individuals)*. Background. Fragrances contain a variety of chemical compounds. Some asthmatics have been reported to have symptomatic responses to some scents. The few available controlled clinical challenge studies had inconsistent findings on respiratory effects of fragrance exposure in sensitive populations, and many studies failed to control for the possible influence of odor perception. Based on the studies that did account for odor, the IOM concluded:

- There is limited or suggestive evidence of an association between exposure to certain fragrances and the manifestation of respiratory symptoms in asthmatics sensitive to such exposures (IOM 2000).

Summary of new evidence. No recent studies were identified.

### Conclusion.

- There is limited or suggestive evidence of an association between exposure to certain fragrances and the manifestation of respiratory symptoms in asthmatics sensitive to such exposures.

### Exposures for which Inadequate or Insufficient Evidence Had Existed to Determine an Association with Exacerbation of Asthma

*Rodent allergens*. Background. Although several mouse and rat allergens have been identified, these may not be the key allergens for all species potentially present in the home (IOM 2000). Work-related allergies to rats or mice are well documented, but clear associations between rodent exposures in homes and exacerbation of asthma had not been established. The IOM concluded:

- There is sufficient evidence of an association between exposure to rodents in a laboratory setting and exacerbation of symptoms or lung function in rodent-sensitized asthmatics.
- There is inadequate or insufficient evidence to determine whether or not an association exists between exposure to rodents (wild or as pets) in the home and exacerbation of symptoms or lung function in rodent-sensitized asthmatics (IOM 2000).

Summary of new evidence. In the Supplemental Material, Table S12 summarizes three available recent studies, all in children: one environmental intervention, one prospective, and one cross-sectional.

Pongracic et al. (2008), in a controlled environmental intervention study that removed mouse allergens from the homes of inner-city children with asthma and sensitization to mouse or rat allergen, did not find a decrease in asthma symptoms or health care utilization to be associated during the intervention year with mouse allergen reduction. They did, however, report significantly reduced school absenteeism, nights of child and caretaker waking, and number of days on which caretakers had to change plans (Pongracic et al. 2008). The study by Pongracic et al. (2008) suggests a potential relationship between mouse allergen exposure in sensitized children and asthma severity or exacerbations. A prospective study on inner-city asthmatic children found that mouse allergen exposure contributed significantly to sensitization, but, among mouse-sensitized children, only to several nonsignificant trends for measures of asthma morbidity (Phipatanakul et al. 2000). Bonner et al. (2006), in a cross-sectional study in children of unknown sensitization status, found no association between presence of rats or mice in the home and symptoms or unscheduled medical visits; however, because sensitization status was unknown, this study does not provide information about responses of rodent-allergic children.

The IOM (2000) had suggested that sufficient home exposures to rodent allergen among sensitized individuals might be expected to be associated with exacerbation of asthma, but insufficient information was available on home exposures and on nonoccupational sensitization. A reconsideration of evidence previously considered by the IOM (2000) on occupational rodent exposure, which demonstrates strong, consistent, temporally appropriate, and biologically plausible relationships, leads to a conclusion of a causal relationship between occupational rodent exposure and exacerbation of asthma in sensitized adult workers. Also, recent evidence shows a substantial minority of inner-city homes to have rodent allergen levels comparable with occupational settings, and also to have rodent-sensitized children (e.g., Matsui et al. 2005).

The two available studies on asthmatic response to rodent allergen among sensitized children, a controlled intervention (Pongracic et al. 2008) and a prospective study (Phipatanakul et al. 2000), found either significant effects for several but not other outcomes, or small and not statistically significant effects in only a few outcomes. These findings do not suggest a clear association. However, together with a determination that occupational rodent exposure causes exacerbation of asthma in sensitized adult workers, and the recent findings about home exposure levels, these findings suggest possible associations in populations of highly exposed, sensitized children.

### Conclusions.

- There is sufficient evidence of a causal association between exposure to rodents in a laboratory setting and exacerbation of symptoms or lung function in rodent-sensitized asthmatics.
- There is limited or suggestive evidence of an association between exposure to rats or mice in the home and increased exacerbation or severity of asthma in rodent-sensitized asthmatic children.

*Cow and horse allergens (acute, nonoccupational exposures)*. Background. Although occupational allergy to cows or horses has been well recognized, data for nonoccupational exposures have not been not readily available. In contrast, evidence has increasingly shown that living on farms with animals may protect children against development of atopy and atopic asthma (IOM 2000). The IOM concluded:

- There is inadequate or insufficient evidence to determine whether or not an association exists between cow or horse allergen in the home and the exacerbation of asthma in sensitive children (IOM 2000).

Summary of new evidence. No recent studies were identified.

### Conclusion.

- There is inadequate or insufficient evidence to determine whether or not an association exists between cow or horse allergen in the home and the exacerbation of asthma in sensitive children.

*Endotoxins*. Background. Endotoxins are compounds found in the outer membranes of gram-negative bacteria. These bacteria are associated with the presence of pets, rodents, and dampness or mold in homes. Toxic effects associated with endotoxins are considered to come from a specific endotoxin, lipopolysaccharide (LPS). Toxicologic and epidemiologic studies have demonstrated that endotoxins cause inflammatory and atopic responses in nonasthmatic and asthmatic subjects, but are also associated with decreased atopy. Variations in the structure of the polysaccharide chain or its lipid portion in different bacteria, the route of exposure, dose rate, age at exposure, and atopic status all potentially influence the biological effects. Adverse effects from endotoxins were apparently increased by other dampness-associated agents, and vice versa (IOM 2000). Because the available evidence supported predictions of either increased or decreased inflammatory responses in asthmatic individuals exposed to endotoxins, the IOM concluded::

- There is inadequate or insufficient information to determine whether or not an association exists between low-level indoor endotoxin exposure and asthma exacerbation (IOM 2000).

Summary of new evidence. In the Supplemental Material, Table S13 describes three recent studies available on endotoxins and exacerbation of asthma: in children, one cross-sectional study; in adults, one controlled challenge study; and in adults and children, one cross-sectional study. Two studies provide evidence associating elevated endotoxin levels with increased asthma severity and bronchial hyperresponsiveness (Rabinovitch et al. 2005; Thorne et al. 2005). Rabinovitch et al. (2005) measured personal daily endotoxin exposures of children in particulate fractions of PM ≤ 2.5 μm diameter (PM_2.5_) and ≤ 10 μm diameter (PM_10_). Endotoxin levels were related to clinically significant increases in asthma severity indices. Personal endotoxin exposures had a significant positive association with asthma symptom scores and with evening FEV_1_, but not with morning FEV_1_. Thorne et al. (2005) reported that endotoxin in bedroom floor dust was associated with significantly elevated ORs for asthma symptoms, asthma medication use, and wheezing; similar but lower associations were found for bedding endotoxin concentrations. No association of increased hay fever risk or protection was found. These effects of endotoxins were found in adults, but not children. The authors concluded that U.S. household endotoxin exposures are associated with asthma symptoms, current asthma medication use, and wheezing, but not with allergy (Thorne et al. 2005). Kitz et al. (2006) found that controlled inhalation challenge to LPS in asthmatic adults caused a significant fall in FEV_1_ 90 min later, reaching a maximum after 120 min.

Among studies cited by the IOM (2000), two provided prior evidence linking indoor endotoxin exposures to exacerbation of asthma: Experimental endotoxin exposure increased bronchial responsiveness to histamine among asthmatic adults, lasting 5 hr, but not among nonasthmatic adults (Michel et al. 1989); a cross-sectional study showed that endotoxin content of house dust was associated with increased asthma severity in dust mite–sensitized adults, but concentration of dust mite allergen was not (Michel et al. 1996). Overall, the evidence suggests that endotoxin exposure is associated with increased inflammation and asthma severity, but not increased allergy. In fact, a growing number of studies suggest that early-life microbiologic exposures to endotoxin may protect against later atopy (e.g., Illi et al. 2014; Lawson et al. 2012), although these exposures are also associated with increased wheeze (Celedon et al. 2007). This potentially protective effect is consistent with the “hygiene hypothesis,” which postulates that growing up in a microbially more hygienic and less diverse environment may increase risk of developing respiratory allergies (Heederik and von Mutius 2012). In contrast, damp or moldy buildings, even though they are associated with increased endotoxin, seem only to increase, not decrease, the development of respiratory disease, even in infants (Mendell et al. 2011).

The available evidence shows a clear association of indoor endotoxin exposure with exacerbation among asthmatic individuals, regardless of whether early exposures prevent later development of allergies. With strong and consistent effects shown in epidemiologic studies and controlled exposures, biologic plausibility for inflammatory effects, and appropriate temporal relationships, the evidence is close to documenting a causal relationship between endotoxin and exacerbation in asthmatics. However, the dual protective and adverse effects suggest caution in labeling indoor endotoxin simply as an adverse causal exposure. The roles of timing, dose, and circumstance of this protective response to exposure need to be better delineated.

### Conclusion.

- There is sufficient evidence of an association between indoor endotoxin exposure and exacerbation of asthma.

*Houseplants*. Background. In theory, houseplants have the potential to release pollen, sap, and other plant parts, or to host pests or fungi that could release allergens, thus provoking allergic responses in people with the necessary sensitization (IOM 2000). Because no studies had been conducted to document these relationships, the IOM concluded:

- There is inadequate or insufficient evidence to determine whether or not an association exists between exposures from houseplants and the exacerbation of asthma (IOM 2000).

Summary of new evidence. No recent studies have addressed houseplant exposure.

### Conclusion.

- There is inadequate or insufficient evidence to determine whether or not an association exists between exposures from houseplants and the exacerbation of asthma.

*Pesticides*. Background. Pesticides include many kinds of fungicides, herbicides, insecticides, and rodenticides. Pesticides are reportedly used in > 80% of U.S. homes. Inhalation and dermal absorption are two potential routes of exposure to pesticides used in the home. The diversity of pesticides used in homes suggests that their relationships to exacerbation of asthma will not be uniform (IOM 2000). The IOM concluded:

- There is inadequate or insufficient evidence to determine whether or not an association exists between pesticide exposures at the levels typically encountered in non-occupational or residential settings and the exacerbation of asthma (IOM 2000).

Summary of new evidence. No recent data have directly addressed pesticides in relation to exacerbation of asthma. In a longitudinal study in the Center for the Health Assessment of Mothers and Children of Salinas (CHAMACOS), increased T helper 2, asthma, and wheeze outcomes in children were associated with reported maternal work in agriculture, which may be an indirect indicator of pesticide exposure to children, either prenatally or at home (Duramad et al. 2006).

### Conclusion.

- There is inadequate or insufficient evidence to determine whether or not an association exists between pesticide exposures at the levels typically encountered in nonoccupational or residential settings and the exacerbation of asthma.

*Plasticizers*. Background. Plasticizers are chemicals that increase the flexibility of plastic resins. A major use of plasticizers is in polyvinyl chloride, widely used indoors in sheet vinyl flooring, wall coverings, vinyl upholstery, and shower curtains. These products are major sources of plasticizer residues, such as di(2-ethylhexyl) phthalate (DEHP) in homes (IOM 2000). DEHP was associated with airway inflammation and possibly with asthma causation. Occupational exposure to plasticizers at high levels was also associated with exacerbation of asthma and asthma causation. The available evidence was very limited. The IOM review concluded

- There is inadequate or insufficient evidence to determine whether or not an association exists between nonoccupational exposure to plasticizers and the exacerbation of asthma (IOM 2000).

Summary of new evidence. Recent studies have demonstrated associations between the presence of plastic materials in homes and increased allergies, respiratory symptoms, and diagnosed asthma (Jaakkola and Knight 2008; Jaakkola et al. 2000, 2006; Mendell 2007), but have not evaluated effects on exacerbation of asthma.

### Conclusion.

- There is inadequate or insufficient evidence to determine whether or not an association exists between nonoccupational exposure to plasticizers and the exacerbation of asthma.

*VOCs other than formaldehyde*. Background. Personal exposures to VOCs are dominated by indoor exposures, even in areas near major outside VOC sources. Hundreds of VOCs have been measured in indoor air, and their diversity suggests lack of uniformity in their potential associations with health. Buildings contain numerous sources of VOCs, including tobacco smoke, combustion appliances, building and renovation materials, house cleaning and maintenance products, solvents, photocopying machines, dry-cleaned clothes, personal care products, printed materials, room deodorizers, moth crystals, and chlorinated water. The main factors influencing exposures are the presence of materials, emission rates, ventilation, and personal behavior (IOM 2000). Very limited evidence suggested any associations between exposures to VOCs indoors and exacerbation of asthma. The multiple correlations between the few studied compounds and many others, often unmeasured, made it difficult to implicate specific VOCs. The IOM review concluded

- There is inadequate or insufficient evidence to determine whether or not an association exists between indoor residential VOC exposures and the exacerbation of asthma (IOM 2000).

Summary of new evidence. Although some studies have suggested associations between indoor VOCs or semi-VOCs and respiratory or allergic effects (Arif and Shah 2007; Choi et al. 2010; Mendell 2007; Rumchev et al. 2004), and between outdoor VOCs and exacerbation of asthma (Delfino et al. 2003), no new evidence related to exacerbation of asthma by indoor VOCs other than formaldehyde was identified.

### Conclusion.

- There is inadequate or insufficient evidence to determine whether or not an association exists between indoor residential VOC exposures (other than formaldehyde) and exacerbation of asthma.

*Pollen exposures indoors*. Background. The primary source of indoor pollen is infiltration from outdoors. Evidence of outdoor pollen in indoor dust samples suggests that it may be an important exposure for exacerbation of asthma (IOM 2000). Because of lack of any direct evidence, the IOM concluded:

- There is inadequate or insufficient evidence to determine whether or not an association exists between pollen exposure in the indoor environment and the exacerbation of asthma (IOM 2000).

Summary of new evidence. No recent evidence regarding indoor pollen exposure and exacerbation of asthma was identified.

### Conclusion.

- There is inadequate or insufficient evidence to determine whether or not an association exists between pollen exposure in the indoor environment and the exacerbation of asthma.

*Down/feather bedding/synthetic bedding*. Background. This question was considered by the IOM (2000) in the context of bird-related allergy. Down bedding, compared with synthetic, was believed to exacerbate asthma because it harbored bird mites. The possibility was also considered that the increased wheeze reported among children using synthetic pillows resulted from increased mite exposure from synthetic pillows (IOM 2000). The IOM concluded:

- There is inadequate or insufficient evidence to determine whether or not an association exists between down pillows and the exacerbation of symptoms or lung function in asthmatics. Down pillows are believed to be a risk factor for asthma because of their documented mite content, rather than because of the presence of bird allergen (IOM 2000).

Summary of new evidence. Glasgow et al. (2011) reported that dust mite–sensitized asthmatic children who used new feather pillows and quilts had nonsignificant reductions in dust mite antigen exposures and wheeze outcomes. Many other studies, reviewed by Siebers and Crane (2011), have shown that feather pillows or quilts, compared with synthetic bedding, accumulate substantially lower concentrations of dust mite antigen and are consistently protective against many respiratory and allergic outcomes, including asthma in unselected populations of infants or children; however, evidence was not available on exacerbation of existing asthma.

### Conclusion.

- There is limited or suggestive evidence that down/feather bedding may be protective for various respiratory effects relative to synthetic bedding, presumably due to lower content of dust mites, although evidence is not available regarding exacerbation of asthma.

*Low outdoor air ventilation rates*. Background. Low outdoor air ventilation rates in buildings lead to increased indoor concentrations of pollutants emitted indoors by occupants, furnishings, equipment, coatings and glues, cleaning products, and building materials. The IOM (2000) considered evidence on whether ventilation rates influenced indoor exposures, and concluded that low ventilation rates strongly influence the level of many indoor exposures, including potential asthma triggers (IOM 2000). Regarding health effects, the IOM concluded:

- Existing data are inadequate for conclusions regarding the association between ventilation rates or ventilation system microbiologic contamination and the exacerbation of asthma symptoms. However, both theoretical evidence and limited empirical data indicate that feasible modifications in ventilation rates can decrease or increase indoor concentrations of some indoor generated pollutants associated with asthma by ≤ 75% (IOM 2000).

Summary of new evidence. A substantial body of research, reviewed by Seppänen et al. (1999) suggests that lower ventilation rates in buildings are associated with a variety of symptoms, including upper and lower respiratory tract symptoms. The effect may be mediated by transmission of respiratory infections or by increased indoor humidity, leading to increased dust mites, fungi, and other microorganisms indoors (Seppänen et al. 1999). No studies were identified that explicitly examine this relationship to exacerbation of asthma. The intervention study by Burr et al. (2007) was considered ineligible because its intervention included increased outdoor air ventilation and mold removal, so study benefits were not specific to increased ventilation.

### Conclusion.

- There is insufficient evidence to determine whether an association exists between lower ventilation rates in buildings and exacerbations of asthma.

## Discussion

This review updates previous conclusions (IOM 2000) with recent evidence, elevating the strength of evidence for some exposures (Table 1). Major changed conclusions include a documented causal relationship with exacerbation for indoor dampness or dampness-related agents (in children); documented associations with exacerbation for dampness or dampness-related agents (in adults), ETS (in preschool-age children), and endotoxin; and limited or suggestive evidence of association with exacerbation for indoor culturable airborne *Penicillium* or total fungi, NO_2_, rodents (nonoccupational exposure), and feather/down pillows (protective relative to synthetic bedding). There is also limited or suggestive evidence that dust mite, cockroach, dog, and dampness-related agents may exacerbate asthma even in nonsensitized individuals, suggesting proinflammatory effects.

In this review we identified limited recent evidence suggesting associations between quantified indoor culturable *Penicillium* or total fungi and exacerbation of asthma. If these associations are confirmed in future studies, this will call into question conclusions based on previous evidence—that available indoor microbial measurement strategies, especially culture-based assays of air samples, are not informative regarding indoor mold-related health effects (Mendell et al. 2011; World Health Organization 2009). Earlier reviews had found no consistent associations between respiratory or allergic health outcomes and quantitative microbial measurements. For instance, few studies had reported significant positive associations between indoor airborne culturable *Penicillium* and specific health outcomes (and these did not include exacerbation of asthma): current asthma in adults (Dharmage et al. 2001); both wheeze and persistent cough in infants, with dose–response relationships (Gent et al. 2002); and respiratory infections in infants (Müller et al. 2002; Stark et al. 2003). No associations were seen between indoor *Penicillium* and allergy (Dharmage et al. 2001) or allergic rhinitis (Stark et al. 2005).

Specific dampness-related causal agents for exacerbation of asthma may include biologic exposures in addition to fungi, such as bacteria, amoebas, and dust mites, or nonbiologic exposures such as chemicals emitted from damp materials (Mendell et al. 2011) [e.g., formaldehyde (McGwin et al. 2010) and 2-ethyl-1-hexanol (Norbäck et al. 2000)]. Furthermore, dampness is associated with respiratory infections (Fisk et al. 2010), the most common cause of exacerbation of asthma (Jackson and Johnston 2010).

Findings from interventional trials of single or multiple strategies, although the strongest study design for demonstrating causality, have often been inconsistent regarding successful reduction of exposures or exacerbation of asthma. A prior review has found that sufficient evidence is available to recommend three types of intervention to reduce asthma symptoms and possibly exacerbations: *a*) tailored in-home education and remediation of asthma triggers, *b*) integrated pest management, and *c*) combined elimination of moisture intrusion or leaks and removal of moldy items (Krieger et al. 2010). Additional rigorous research is needed to better document causality and determine the effectiveness of specific remediation strategies (Sauni et al. 2011).

This review did not include the indoor intrusion of outdoor pollutants, including ozone, SO_2_, NO_2_, fungal spores, pollen, and nonbiologic PM such as diesel exhaust particles. Outdoor exposures to PM are associated with asthmatic symptoms (Delfino et al. 2002; Rabinovitch et al. 2006; Sheppard et al. 1999) and exacerbations (Chang et al. 2009; Salam et al. 2008; Spira-Cohen et al. 2011). Experimental exposures to SO_2_ and ozone result in exacerbation of asthma, airway inflammation, and increased response to inhaled allergens (Linn and Gong 1999; Peden et al. 1995). Some strategies for reducing pollutant exposures indoors may also reduce indoor exposures to outdoor-generated pollutants. Such strategies might include using air conditioning and high-efficiency particulate filters in home mechanical systems, and thorough daily washing to remove allergens settled on people (Diette et al. 2008). However, reducing entry of outdoor pollutants by keeping doors and windows closed or sealing a building against air infiltration would result in increased indoor concentrations of indoor-generated pollutants, unless effectively enhanced particle filtration or gas air cleaning strategies were used. In contrast, increasing ventilation rate to dilute indoor-generated pollutants may increase indoor concentrations of outdoor pollutants. The advantages and disadvantages of ventilation-related strategies for controlling indoor exposures must be weighed, considering the type and amounts of specific indoor and outdoor pollutants. For a more detailed discussion of the impact of ventilation on indoor exposures relevant to asthma, see Chapter 10 of the IOM review (IOM 2000).

This review builds on the conclusions of the IOM (2000) document without thoroughly reexamining prior evidence and without inclusion of unpublished studies, and therefore is limited in its scope and potentially subject to publication bias. A further limitation is the lack of uniformity in reviewed studies of the definitions for exacerbation of asthma and the tools for assessing exacerbation. In addition, the literature used in this review was restricted to findings published in English. Therefore, conclusions drawn from this review should be considered provisional.

In future research, the inconsistencies in current research may be reduced by improved exposure assessment, such as for dampness-related microbial agents (e.g., non–culture-based, species-specific assays) and determination of subjects’ sensitization. More rigorous study designs should be emphasized: performing cross-sectional studies only for hypothesis generation; using intervention, prospective, or true nested case–control designs to confirm associations; and conducting rigorously designed intervention studies with careful measurements of exposures and health to document the causality and effectiveness of real-world environmental interventions.

## Conclusions

In this review we have revised prior evidence-based conclusions about relationships between specific indoor exposures and exacerbation of asthma. Exposures to indoor dampness and dampness-related agents have a causal relationship with exacerbation of asthma (in children). Exposures to dampness-related agents (in adults), ETS (in preschool-age children), and endotoxin are associated with exacerbation of asthma. Exposures to indoor culturable *Penicillium* and total fungi, rodents (nonoccupational exposure), and NO_2_ have limited or suggestive evidence for an association with exacerbation of asthma, and limited data suggest that exposures to feather/down pillows may have a protective association relative to synthetic bedding. Exposures to dust mite, cockroach, dog, fungi, and dampness-related agents also have limited or suggestive evidence for an association with exacerbation of asthma even in nonsensitized individuals, suggesting proinflammatory effects. Prospective or intervention studies are needed to confirm hypothesized associations, and rigorous real-world environmental intervention trials are needed to demonstrate effective remediation and resulting reductions in exacerbation of asthma.

## Supplemental Material

(1.1 MB) PDF
