# Supplementary material for: Indoor Environmental Exposures and Exacerbation of Asthma: An Update to the 2000 Review by the Institute of Medicine
Source: Environ Health Perspect. 2014 Oct 10;123(1):6–20. doi: 10.1289/ehp.1307922 (PMC4286274; doi:10.1289/ehp.1307922)
Supplement: (1.1 MB) PDF [file ehp.1307922.s001.508.pdf]

Environmental Health Perspectives doi:10.1289/ehp.1307922

Supplemental Material

Indoor Environmental Exposures and Exacerbation of Asthma: An Update to the 2000 Review by the Institute of Medicine

Watcharoot Kanchongkittiphon, Mark J. Mendell, Jonathan M. Gaffin, Grace Wang, and Wanda Phipatanakul

Table S1. Categories of evidence (IOM 2000).

**Level of Evidence**	**Definition**
Sufficient Evidence of a Causal Relationship	Evidence is sufficient to conclude that a causal relationship exists between the action or agent and the outcome. That is, the evidence fulfills the criteria for "Sufficient Evidence of an Association" below and in addition satisfies criteria used to assess causality, regarding the strength of association, biologic gradient (dose–response effect), consistency of association, biologic plausibility and coherence, and temporality.
Sufficient Evidence of an Association	Evidence is sufficient to conclude that there is an association. That is, an association between the action or agent and the outcome has been observed in studies in which chance, bias, and confounding can be ruled out with reasonable confidence. For example, if several small studies that are free from bias and confounding show an association that is consistent in magnitude and direction, there may be sufficient evidence of an association.
Limited or Suggestive Evidence of an Association	Evidence is suggestive of an association between the action or agent and the outcome but is limited because chance, bias, and confounding cannot be ruled out with confidence. For example, at least one high-quality study shows a positive association, but the results of other studies are inconsistent.
Inadequate or Insufficient Evidence to Determine Whether or Not an Association Exists	The available studies are of insufficient quality, consistency, or statistical power to permit a conclusion regarding the presence or absence of an association; or no studies exist that examine the relationship. For example, available studies have failed to adequately control for confounding or have inadequate exposure assessment.
Limited or Suggestive Evidence of No Association	Several adequate studies are mutually consistent in not showing an association between the action or agent and the outcome. A conclusion of "no association" is inevitably limited to the conditions, level of exposure, and length of observation covered by the available studies. In addition, the possibility of a very small elevation in risk at the levels of exposure studied can never be excluded.

Study inclusion criteria

The following inclusion and exclusion criteria were applied to published study.

Inclusion criteria

1. Primary or secondary objective of study is to examine risk factors for asthma.
2. Outcome is exacerbation or improvement of asthma that was previously diagnosed.
3. Independent variable of interest is an environmental trigger or risk factor.
4. Empirical study using one of the following designs:
    a. Observational case series
    b. Observational cross sectional
    c. Observational case control
    d. Observational retrospective or prospective cohort
    e. Experimental or intervention-based
    f. Meta-analysis

Exclusion criteria

1. Outcome is
    a. incident asthma, new development of asthma, or prevalence of asthma (i.e., not symptom worsening or exacerbation of diagnosed asthma)
    b. any other respiratory condition or symptom (e.g., wheezing alone without diagnosis of asthma)
    c. other (e.g., lung function)
2. Primary study objective is to examine exacerbation or improvement of asthma associated with:
    a. smoking
    b. obesity/BMI
    c. exercise

d. co-morbid conditions
e. medications, drug, or substance
f. socioeconomic status
g. psychosocial factors
h. food, food allergies
i. other
j. infections
k. occupational exposures
l. sensitization

3. Study objective is to examine management program, health service efficacy/effectiveness.
4. Study examines cellular function, biological mechanism, or genetic associations, in absence of environmental trigger or risk factor.
5. The environmental risk factor is used only or primarily as an adjustment factor.
6. The study design is:
    a. Prevalence study
    b. Clinical trial
    c. Qualitative study
    d. Case report
    e. Validation study of measurement tools
    f. Economic evaluation
    g. Review (non systematic)
    h. Expert opinion, commentaries, letters, etc.
7. Study focuses on outdoor environment, including allergens and ambient air pollutants.
8. There is no mention or discussion of asthma in abstract or the study population does not include people with asthma.

9. Focus is on validation or measurement of biomarkers, e.g., exhaled nitric oxide.

10. Article describes a study protocol.

11. Study based on animal models.

Literature search strategy

PubMed Search 1 conducted on May 27, 2011, "MeSH major topic" as category tags
asthma[major]

AND

("Animals, Domestic"[major] OR "Cockroaches"[major] OR dust[major] OR "Endotoxins"[major] OR "Fungi"[major] OR "Plants"[major] OR "Pollen"[major] OR "Rhinovirus"[major] OR "Chlamydia trachomatis"[major] OR "Chlamydophila pneumoniae"[major] OR "Mycoplasma pneumoniae"[major] OR "Nitrogen Oxides"[major] OR "Pesticides"[major] OR "Ozone"[major] OR "Particulate Matter"[major] OR "Sulfur Oxides"[major] OR "Plasticizers"[major] OR "Volatile Organic Compounds"[major] OR "Formaldehyde"[major] OR "Perfume"[major] OR "Tobacco Smoke Pollution"[major])

NOT

(Editorial[PT] OR Letter[PT] OR Practice Guideline[PT] OR Addresses[PT] OR Autobiography[PT] OR Bibliography[PT] OR Biography[PT] OR Case Reports[PT] OR Clinical Conference[PT] OR Comment[PT] OR Congresses[PT] OR "Consensus Development"[PT] OR Dictionary[PT] OR Directory[PT] OR In Vitro[PT] OR Interactive Tutorial[PT] OR Interview[PT] OR Lectures[PT] OR Legal Cases[PT] OR Legislation[PT] OR News[PT] OR Newspaper Article[PT] OR Patient Education Handout[PT] OR Portraits[PT] OR Video-Audio Media[PT] OR Webcasts[PT] OR "Clinical Trial, Phase I"[PT] OR "Clinical Trial, Phase II"[PT] OR "Clinical Trial, Phase III"[PT] OR "Clinical Trial, Phase IV"[PT]) OR ("genetic Predisposition to Disease"[major] OR genome[ti] OR *genetic*[ti] OR polymorphism[ti])

Limits: only items with abstracts, Humans, English, published in the last 10 years

Hits: 1,225

PubMed Search 2 conducted on August 4, 2011, "MeSH terms" as category tags

asthma[major]

AND

("Animals, Domestic"[mesh] OR "Cockroaches"[mesh] OR dust[mesh] OR "Endotoxins"[mesh] OR "Fungi"[mesh] OR "Plants"[mesh] OR "Pollen"[mesh] OR "Rhinovirus"[mesh] OR "Chla-

mydia trachomatis"[mesh] OR "Chlamydophila pneumoniae"[mesh] OR "Mycoplasma pneumoniae"[mesh] OR "Nitrogen Oxides"[mesh] OR "Pesticides"[mesh] OR "Ozone"[mesh] OR "Particulate Matter"[mesh] OR "Sulfur Oxides"[mesh] OR "Plasticizers"[mesh] OR "Volatile Organic Compounds"[mesh] OR "Formaldehyde"[mesh] OR "Perfume"[mesh] OR "Tobacco Smoke Pollution"[mesh])

NOT

(Editorial[PT] OR Letter[PT] OR Practice Guideline[PT] OR Addresses[PT] OR Autobiography[PT] OR Bibliography[PT] OR Biography[PT] OR Case Reports[PT] OR Clinical Conference[PT] OR Comment[PT] OR Congresses[PT] OR "Consensus Development"[PT] OR Dictionary[PT] OR Directory[PT] OR In Vitro[PT] OR Interactive Tutorial[PT] OR Interview[PT] OR Lectures[PT] OR Legal Cases[PT] OR Legislation[PT] OR News[PT] OR Newspaper Article[PT] OR Patient Education Handout[PT] OR Portraits[PT] OR Video-Audio Media[PT] OR Webcasts[PT] OR "Clinical Trial, Phase I"[PT] OR "Clinical Trial, Phase II"[PT] OR "Clinical Trial, Phase III"[PT] OR "Clinical Trial, Phase IV"[PT]) OR ("genetic Predisposition to Disease"[major] OR genome[ti] OR *genetic*[ti] OR polymorphism[ti])

Limits: only items with abstracts, Humans, English, published in the last 10 years

After omitting articles from Pubmed Search 1 (above), hits: 871

PubMed Search 3 conducted on August 5, 2013

The same strategy as the 1st and 2nd search was used. 429 abstracts were retrieved using "MeSH terms" as a category tag and 293 abstracts were retrieved using "MeSH major topic" as a category tag. This search was restricted to the restricted to publication published from May and August 2011.

Prior evidence for selected exposures (IOM 2000)

House dust mite allergens

The evidence considered by the IOM (2000) in evaluating the factors supporting causality included:

- Strength and Consistency of association – Many studies showed that dust mite sensitization was strongly associated with asthma (Gelber et al., 1993; Peat et al., 1996; Squillace et al., 1997; Sears et al., 1989; Sporik et al., 1990). However, these did not document a direct association of allergen exposure to asthma morbidity. Dust mite bronchial challenge by Cockcroft et al. (1979) in sensitized individuals caused an immediate fall in FEV_1. A number of intervention studies have shown that environmental changes that reduced exposure of asthmatic individuals to dust mite allergen resulted in decreased asth-

ma symptoms and decreased bronchial hyperreactivity (Platts Mills and de Weck 1988). However, these interventions studies, which also included reduction in other exposures and changes in other factors, were not specific to dust mite allergen reduction and thus were not sufficient to demonstrate causality or even unconfounded association.

- Biologic gradient/Dose-response - A dose-response relationship between dust mite exposure and sensitization development was demonstrated by Kuehr et al. (1994) with the threshold concentration of 2 ug/g. But, this study did not demonstrate a dose-response for asthma morbidity among those with asthma.
- Biologic plausibility and coherence - Dust mite bronchial challenge by Cockcroft et al. (1979) in sensitized individuals caused an immediately fall in FEV_1. After challenge, eosinophils were recruited in to the lungs and might persist for up to two weeks (Shaver et al. 1997).
- Temporally correct association - Mite allergic children who moved to very low dust mite environments were shown to have clinical improvement (Piacentini et al. 1996). However, in this and other similar studies, there might have been other changes associated with the move to mite-free environment (e.g., no animals, very low spore count).

Cat allergens

The evidence cited by the IOM (2000) in evaluating the factors supporting causality included:

- Strength and consistency of association – Cat allergen can induce allergic symptoms, asthmatic symptoms, and decreased lung function in cat sensitized patients. Norman et al. (1996) demonstrated that exposure to inhaled cat allergen in an experimental cat room led to decreased FEV_1, with magnitudes comparable to the response to inhalation challenge with cat allergen in a similar population (Sicherer et al. 1997). Norman et al. (1996) documented increasing nasal and lung symptoms scores during 60 minutes in the cat room. Bollinger et al. (1996) demonstrated increased upper- and lower-respiratory symptoms and decrements in lung function even at levels of cat allergen occurring in homes without cats. in a double-blind, placebo-controlled, cross-over study of twenty asthmatic children sensitized to cat or dog allergens, and living in homes with these animals, airway hyperresponsiveness was improved and peak flow variation was decreased during the use of air cleaners in the living room and bedroom of the child (van der Heide et al., 1999).

- Biologic gradient/Dose-response - Dose-response relationships for cat allergen were not cited.
- Biologic plausibility and coherence - *Fel d* 1 has been identified as the major cat allergen. High levels of *Fel d* 1 are found in the air and dust of homes with cats, but are also found in many buildings without resident cats (IOM 2000). Upper and lower respiratory response to this allergen among those sensitized has clear plausability.
- Temporally correct association - No studies are available that evaluate symptoms or lung function in cat-sensitized asthmatics before and after removal of the cat from the home.

Nor are there studies of change in symptoms or lung function after moving from a home with a cat to a home without a cat. However, exposure studies suggest that in cat-sensitive subjects the decline in lung function associated with low-level airborne exposure to cat allergen, which can be present in a home with no cats, tends to be less extreme than the decline in lung function associated with higher-level airborne exposure (Bollinger et al., 1996). The experiments demonstrating entry into a room with a cat as a source of exacerbation of asthma in cat-sensitive individuals also suggest that removal of the cat from the household may decrease symptoms in cat-allergic asthmatic. Still, even if the cat is removed from the home, continued low-grade exposures may occur in public places or via clothes from cat owners. Since cat allergen is everywhere, there is little potential for absolute avoidance (Dybendal and Elsayed, 1994; Warner, 1992).

The mix of prior findings in studies of cat allergens, atopy, and asthma was complex, suggesting possibly different effects depending on age at exposure, level of exposure, and sensitization status. Early exposure to cats may prevent sensitization to cat allergens in some children, although findings have not been fully consistent.

Cockroach allergens

The evidence considered by the IOM (2000) in evaluating factors supporting causality included:

- Strength of association ---Association of exposure to allergen and asthma morbidity was shown by Rosenstreich et al. 1997, in a prospective study that found over three times the rate of hospitalization in exposed sensitized children. Also, two controlled challenge studies documented a strong association in adults, clearly causal. Bernton et al. (1972) and Kang (1976) documented that controlled challenges to cockroach extract in cockroach-sensitive asthmatic subjects provoked an immediate asthmatic reaction, including acute and late bronchospasm and antigen-specific eosinophilia. Antigen-induced asthmatic reactions were blocked by premedication with disodium cromoglycate (Kang 1976).

- Biologic gradient/Dose-response -- A dose-response between cockroach allergen exposure and development of specific sensitization was reported to have been shown (Sarpong et al. 1996; Eggleston et al. 1998; Sarpong and Han. 1999). A dose-response relation was said to be demonstrated between cockroach allergen exposure in bedrooms and asthma morbidity in specifically sensitized children (Rosenstreich et al. 1997). (Note: The three studies showing dose-response between cockroach allergen exposure and sensitization do not demonstrate a dose-response for asthma morbidity. Also, Rosenstreich et al. (1997) did not in fact show a dose-response, but only compared a single higher level to a reference level to find statistical significance for hospitalizations, unscheduled medical visits for asthma, missed school days, nights with lost sleep, days of wheezing, awakening of caregivers and changed plans of caregivers. Thus while the well-designed study by Rosenstreich et al. (1997) showed strength of association, dose-response for cockroach

allergen and asthma morbidity in sensitized was not demonstrated by this or other prior data.)

- Consistency of association -- The degree of allergen exposure has been clearly related to the development of specific sensitization, and sensitization is related to asthma morbidity (Eggleston et al., 1998; Rosenstreich et al., 1997; Sarpong and Han, 1999; Sarpong et al., 1996a; Sastre et al., 1996). In a study not restricted to diagnosed asthmatics, Gold et al. (1999) found in a prospective study that infants born in homes with high levels of cockroach allergen had increased risk of wheezing by the age of 1 year. The association of cockroach allergen exposure with new onset asthma (Litonjua et al. 1997) was considered to be consistent with these exposures causing exacerbation.

- Biologic plausibility and coherence -- Bernton et al. (1972) and Kang (1976) documented that controlled challenges to cockroach extract in cockroach-sensitive adult asthmatic subjects provoked an immediate asthmatic reaction, including acute and late bronchospasm and antigen-specific eosinophilia. Antigen-induced asthmatic reactions were blocked by premedication with disodium cromoglycate (Kang 1976). (Note: Both studies found effects only in asthmatics with specific sensitivity, documenting effects occurring exclusively through specific allergic response.)

- Temporally correct association – (Note: Temporality of association, although not specifically discussed in IOM (2000), was apparent in the cited challenge studies in adults and the prospective study in children.)

The IOM (2000) concluded that "available research demonstrates associations of cockroach antigen exposure in infants with increased specific sensitization to a degree related to extent of exposure, and association of exposure in infants with wheeze, *but does not document asthma exacerbation per se as a result of the exposure . . . it is difficult to predict whether cockroach allergen per se is an initiator of asthmatic symptoms*," *[emphasis added]* but overall, "it is the combination of strong association, biological plausibility, dose–response, and provocation experiments that creates the strength of the argument."

Environmental tobacco smoke exposures

The evidence considered by the IOM (2000) in evaluating factors supporting causality included:

- Strength and consistency of association – A broad review by Cook and Strachan (1999) concluded that "among children with established asthma, parental smoking was associated with more severe disease." The IOM (2000) concluded that, based on the available evidence "ETS exposure is associated with more frequent asthma exacerbations" in children, when considering chronic exposure. Since most exposed individuals are likely to have chronic exposure, assessing the effect of acute ETS exposure to exacerbation of asthmais difficult. ETS exposure in a chamber was investigated in adults with asthma. In

adults, one study correlated asthma symptoms with reported daily ETS exposure and reported greater odds for restricted activity days in relation to ETS exposure level, with a higher ratio for the level of asthma symptoms associated with having a smoker in the home, suggesting an effect of chronic as well as acute ETS exposure (Ostro et al., 1994). Some studies assessed ETS exposure in chambers in adults. These studies, which were reviewed in detail by the California EPA (1997), have shown slight to moderate transient effects on lung function in at least a portion of participants but have not demonstrated a consistent effect.

- Biologic gradient/Dose-response – Limited evidence was available to demonstrate this
- Biologic plausibility and coherence - Smoking was associated with significant structural changes in both airways and the pulmonary parenchyma (U.S. DHHS, 1984), including hypertrophy and hyperplasia of the upper airway mucous glands, leading to an increase in mucous production with associated increased prevalence of cough and phlegm. Chronic inflammation of the smaller airways also occurs, leading to bronchial obstruction. In addition, airway narrowing may occur consequent to destruction of the alveolar walls, decreased lung elasticity, and development of centrilobular emphysema (U.S. EPA,1992). Smoking also may increase mucosal permeability to allergens, increasing total and specific IgE levels (Zetterstrom et al., 1981) and blood eosinophil counts (Halonen et al., 1982).
- Temporally correct association - A reduction in exposure, short of total avoidance to ETS, has shown some benefit (Cook and Strachan, 1999).

Dog allergens

The evidence considered by the IOM (2000) in evaluating factors supporting causality included:

- Strength and consistency of association – A positive bronchial provocation test with dog allergen, in a cross-sectional study of asthmatic children (Vanto and Koivikko, 1983), was correlated with a positive skin prick test to dog, although not with the frequency of reported symptoms. In addition, in immunotherapy trials, a positive response to bronchial provocation with dog allergen has been associated with increased levels of IgE to dog allergen in asthmatic subjects (Hedlin et al., 1995; Valovirta et al., 1984; Vanto et al., 1980). However, there were no reports of responses of dog-sensitized asthmatics to controlled dog allergen exposure or to home exposures in epidemiologic studies (IOM 2000).
- Biologic gradient/Dose-response – There were no published studies of the responses of dog-sensitized asthmatics to different levels of dog allergen exposure.
- Biologic plausibility and coherence – *Two* purified dog allergens have been identified (Schou, 1993). *Can f* I is present in dander, pelt, hair, and saliva of dogs (Schou, 1993). *Can f* I, considered a major allergen, accounts for at least half of the allergenic activity in dog hair and dander. Exacerbation of asthma among dog-sensitized individuals upon exposure to dog allergen is thus biologically plausible.

- Temporally correct association – Correct temporal association is apparent in provocation tests with dog allergen (Vanto and Koivikko, 1983). In a cross-sectional study of asthmatic children, parents tended to report that removal of the dog had improved asthma (Vanto and Koivikko, 1983)

Fungal exposures (quantified)

The IOM review considered fungal exposure primarily as a trigger of asthmatic responses among sensitized individuals. The IOM review summarized the available evidence for fungal exacerbation of allergic asthma, along with some of the difficulties of documenting these relationships. The IOM reported that increased asthma severity with higher outdoor fungal spore concentrations (Delfino et al. 1997; Malling 1986) provocation of asthma symptoms among patients with fungal sensitivities (Lelong et al. 1986; Malling 1986; Licorish et al. 1985), as well as reduction in asthma symptoms by desensitization with fungal antigens (Cantani et al. 1988; Horst et al. 1990; Dreborg et al. 1986) were demonstrated repeatedly.

Links between indoor fungal exposures and exacerbation of asthma were more uncertain and inconsistent. For instance, because fungi are generally present at far greater concentrations outdoors and documenting indoor fungal growth can be challenging, it was difficult to eliminate outdoor exposure as the primary determinant of fungal sensitization and symptoms. The specific exposure parameters leading to fungal exacerbations of asthma were not clear due to inadequate environmental and diagnostic testing procedures, among other factors (IOM 2000).

Nitrogen dioxide

Few available studies had assessed the respiratory effects of indoor NO_2 on asthmatic subjects, thus limiting the IOM's ability to assess the effects of NO_2 on exacerbations of asthma. The existing cross-sectional studies had mixed findings on the respiratory effects of gas stove exposure among general populations of adults or children, with no discernible explanation for the conflicting findings. Findings of studies that also included NO_2 measurements were not more consistent, suggesting either limitations in NO_2 exposure assessment or a primary causal role for other gas stove-associated pollutants. Associations between gas appliance use or NO_2 concentrations and declines in lung function were inconsistent.

Direct effects of NO_2 exposure were most clearly evident in controlled chamber studies, although these studies were limited by small numbers of subjects and short exposure times, often requiring unrealistically high NO_2 concentrations. Studies in asthmatic adults showed that brief, high-level NO_2 exposures, although not causing symptoms directly, enhanced airway responsiveness to specific (e.g., dust mite allergen) and nonspecific (e.g., histamine) challenges at concentrations that might be found in poorly ventilated kitchens during gas appliance use (IOM 2000).

The evidence considered by the IOM (2000) in evaluating the factors supporting causality included:

- o Strength and consistency of association – Chamber studies involving one- to three-hour exposures to 50–1,500 ppb, NO_2 did not find any effect on respiratory symptoms among asthmatic subjects (Salome et al., 1996; Utell et al., 1991). However, studies in asthmatics reported enhanced airway responses to histamine or methacholine challenges at concentrations as low as 500–600 ppb, (Mohsenin, 1987; Salome et al., 1996). Epidemiologic evidence in general populations from many studies on respiratory effects of gas stove use, or of measured NO2, was inconsistent.
- o Biologic gradient/Dose-response – Chamber studies involving one- to three-hour exposures to 50–1,500 ppb, NO_2 did not found any effect on respiratory symptoms among asthmatic subjects (Salome et al., 1996; Utell et al., 1991); thus, supportive evidence was not available.
- o Biologic plausibility and coherence – Studies in asthmatics reported enhanced airway responses to histamine or methacholine challenges at concentrations as low as 500–600 ppb, lower than in normal subjects, suggesting that the airways of asthmatics were more sensitive to the enhancing effects of NO_2 (Mohsenin, 1987; Salome et al., 1996). Temporally correct association – To the extent that controlled exposures enhanced air way responses to other challenges, the correct temporal association was demonstrated.

Rodent allergens (non-occupational)

Work-related allergies to rats or mice had been well documented in many studies, Clear associations between exposure to rats and mice in the home and exacerbation of asthma had not been established. Limited evidence was available to the IOM (2000) in evaluating the following factors supporting causality:

- o Strength and consistency of association – Many studies have shown associations between laboratory exposures to rodents and sensitization, and between sensitization and asthmatic symptoms. A prospective study showed significant associations between working with rats and symptoms and decreased peak flow among those specifically sensitized (Hollander et al., 1996). These are sufficient to demonstrate consistent association in work settings. Although, no studies examined associations between indoor nonoccupational rodent allergen exposure and exacerbation of asthma, the question is one of sufficient dose in those sensitized, not of whether such a relationship exists at all.
- o Biologic gradient/Dose-response –No data were available on multiple rodent allergen levels at work or in the home and dose-related exacerbation of asthma among rodent-sensitized asthmatics.
- o Biologic plausibility and coherence – Rodent hair and epithelial fragments carry allergenic molecules believed to be from urine, saliva, or skin. Allergenic protein from sprayed urine dries up and becomes airborne on dust particles. Airborne rodent allergen has been

measured in laboratory facilities in many studies but in only one study in inner city apartments (Swanson et al., 1985). Clinically important allergens have been identified for mouse, rat, and guinea pig (Schou, 1993). In the U.S. National Cooperative Inner City Asthma Study, 19% of asthmatic children were allergic to rats and 15% were allergic to mice, suggesting exposure to rat or mouse allergens in the home (Kattan et al., 1997). Thus plausible biologic mechanisms have been demonstrated.

- Temporally correct association –At least one prospective study (Hollander et al., 1996) showed significant associations between working with rats and changes in symptoms and peak flow among sensitized workers.

Recent study findings

Table S2. Recent evidence on association of house dust mite allergens with exacerbation of asthma.

Source	Study Design	Measured Exposures	Findings
Children			
(El-Ghitany et al. 2012)	Randomized, controlled intervention	Interventions to reduce dust mite allergen from bedroom	Reduction in dust mite allergen concentrations, by physical or chemical means, were associated with reduced asthma morbidity and severity in sensitized children.
(Halken et al. 2003)	Prospective	Dust mite allergen from bed	Encasing of mattresses and pillows resulted in a significant long-term reduction in HDM allergen concentrations in mattresses and in the need for inhaled steroids in children with asthma and HDM allergy.
(Gent et al. 2012)	Prospective	Dust mite allergen from floor and furniture of the main living area	Dust mite allergen exposures were significantly associated with increased rescue medication use in sensitized children.
(Gent et al. 2009)	Prospective	Dust mite allergen from bedroom and living room	Dust mite allergen concentrations were significantly associated with moderate to severe severity scores and with medication use. Associations were similar regardless of mite sensitization. There was a dose-related positive association between dust mite allergen and wheeze.
(Nitschke et al. 2006)	Prospective	House dust mite allergen in beds	Demonstrated increased risk among sensitized asthmatic children in rates of nighttime wheeze, daytime cough, and daytime asthma attacks in relation to Der p 1 levels in beds greater than 10-ug/g.
(Spanier et al. 2006)	Cross-sectional	Dust mite allergen from bedroom	Dust mite allergen concentrations had a significant positive association with FeNO levels. This association was only slightly, and not significantly, stronger in mite-sensitized children.
(Murray et al. 2006)	Cross-sectional (Case-control)	Dust mite from bed and living room floor	Children in the acute exacerbation group were non-significantly more likely (OR=1.6, 95% CI 0.9-2.9) to be sensitized and exposed to dust mite allergen than those in the stable asthma group.
(Rabito et al. 2011)	Cross-sectional	Dust mite allergen from bedroom	Dust mite allergen concentrations were not significantly associated with any measure of asthma morbidity, such as hospital admission or wheezing, either in sensitized or not sensitized.
(Turyk et al. 2006)	Cross-sectional	Dust mite from bedroom and living room	Dust mite allergen concentrations were not associated with number or frequency of symptoms. Information on mite sensitization was not available.
Adult			
(Dharmage et al. 2006)	Double blinded, randomized controlled trial	Intervention to reduce dust mite allergen from bed	Encasement of bedding significantly reduced the Der p 1 levels. However, this was not sufficient to produce worthwhile clinical improvement in dust-mite sensitized adults.
Adults and Children			
(Langley et al. 2005)	Cross-sectional	Dust mite allergen from living room	Adult atopic asthmatics not sensitized to mite but exposed to high levels of mite allergen had significantly more severe bronchial hyper-responsiveness than subjects not exposed to high levels of mite

Abbreviations: FeNO, fraction exhaled nitric oxide; HDM, house dust mite.

Table S3. Recent evidence for the association of cat allergens with exacerbation of asthma.

Source	Study Design	Measured Exposures	Findings
Children			
(McConnell et al. 2006)	Prospective	Presence of cat	Among children with a cat, there were generally larger effects of air pollution than among those without cats. However, the differences by strata of cat ownership were statistically significant only for organic acid.
(Gent et al. 2012)	Prospective	Cat allergen in dust from floor and furniture of the main living area	Cat allergen exposures were significantly associated with increased rescue medication use in sensitized children.
(Gent et al. 2009)	Prospective	Cat allergen in dust from bedroom and living room	Among cat-sensitized children, cat allergen had a significant positive association with increased asthma severity (30+ days of wheeze, medication use for 9+ months)
(Murray et al. 2006)	Cross-sectional (case-control)	Cat allergen in dust from bed and living room floor	Children in the acute exacerbation group were significantly more likely to be sensitized and exposed to cat allergen than those in the stable asthma
(Spanier et al. 2006)	Cross-sectional	Cat allergen in dust from bedroom	Higher cat allergen exposure and owning a cat were both associated with *lower* FeNO levels
(Turyk et al. 2006)	Cross-sectional	Cat allergen in dust from bedroom and living room	Cat allergens from the living room were not significantly associated with asthma symptoms. Cat allergens from the bedroom were associated with increases, although not statistically significant, in both the number and frequency of asthma symptoms.
Adult and children			
(Langley et al. 2005)	Cross-sectional	Cat allergen from living room	In subjects not sensitised to cat, there was no difference in PD20, eNO, or FEV_1 % predicted between those exposed to high levels of that allergen and those not exposed.

Abbreviations: eNO, exhaled nitric oxide; FeNO, fraction of exhaled nitric oxide; FEV_1, forced expiratory volume in 1 second; PD20, provocation dose causing fall in FEV_1 of at least 20%.

Table S4. Recent evidence on association of cockroach allergens with exacerbation of asthma.

Source	Study Design	Measured Exposures	Findings
Children			
(Gruchalla et al. 2005)	Prospective	Cockroach allergen in dust from bedroom	sensitized children exposed to bedroom cockroach allergen had significantly more asthma symptom days, school days missed, and caretaker interrupted sleep, and nonsignificantly more unscheduled medical visits and hospitalizations, than those not sensitive or exposed.
(Bonner et al. 2006)	Cross-sectional	Cockroach presence, from interview	Reported presence of roaches was not associated significantly with several types of asthma symptoms, unscheduled medical visits, or steroid-treated asthma attacks. No data on individual allergy status.
(Gent et al. 2009)	Cross-sectional	Cockroach allergen in dust from bedroom and living room	Cockroach allergen in living room or bedroom was not associated with asthma severity in asthmatic children, regardless of specific sensitization status.
(Rabito et al. 2011)	Cross-sectional	Cockroach allergen in dust from kitchen	Exposure to cockroach allergens was associated with a 4- to 5-fold increase in hospital admissions of asthmatic children. (This may be the factor driving high rates of asthma morbidity seen in inner-city children.)
(Spanier et al. 2006)	Cross-sectional	Cockroach allergen in dust from bedroom	Cockroach allergen exposure in the bedroom was not associated with ENO level in asthmatic children of unknown atopic status
(Turyk et al. 2006)	Cross-sectional	Cockroach allergen in dust from bedroom and from living room	Cockroach allergen levels in bedrooms of asthmatic children (unknown atopic status) had significant dose-related association with number of symptoms (ORs 1.0, 4.4, 5.8), although respiratory infections confounded this relationship, and nonsignificant doubling of symptom frequency. No significant association found with living room exposure.
Adults and Children			
(Shedd et al. 2007)	Cross-sectional	Presence of cockroaches	A visibly roach-free environment was not significantly associated with improvement in asthma-related quality of life or asthma symptoms in adults.

Abbreviations: ENO, exhaled nitric oxide.

Table S5. Recent evidence on association of environmental tobacco smoke with exacerbation of asthma.

Source	Study Design	Measured Exposures	Findings
Preschool Children			
(Kattan et al. 2007)	Prospective	Urinary cotinine	Higher ETS exposures associated with only a marginally statistically significant decrease in peak flow in cold weather only, and not with differences in wheeze or unscheduled medical visits
(Perzanowski et al. 2010)	Prospective	Survey	Current ETS exposure was significantly *inversely* related to FeNO and previous ETS exposure was significantly *positively* associated with FeNO. (ETS exposure may elevate FeNO and decrease lung function in children over a period of few years.) ETS exposure at age 4 was significantly associated with lower FEV_1 and $FEV_{25–75\%}$.
Children			
(Spanier et al. 2008)	Prospective	Survey, biomarkers of internal dose, and an indoor nicotine dosimeter – (number Of cigarettes smoked per day in the home; hair and serum cotinine; dosimeter-measured tobacco smoke in home)	FeENO not associated with reported tobacco smoke exposure, serum cotinine, or hair cotinine. Measured nicotine exposure was associated with *decreased* FeNO.
(Spanier et al. 2009)	Prospective genetic association study	See Spanier 2008, above	The study explored genetic interactions as explanations for the lack of prior associations between ETS indicators and FeNO. Among tobacco smoke exposed children with asthma, found polymorphisms with which ETS was not associated with FeNO, and several polymorphisms with which ETS exposures were associated with decreased FeNO.
(Soussan et al. 2003)	Prospective	Survey	Symptom control was inversely related to smoking in the home. PEF was less achieved if the mother smoked.
(Lawson et al. 2011)	Case-control	Saliva cotinine levels	Tobacco smoke exposure was associated with increased risk of high diurnal-variation in PEF among female children
(Chapman et al. 2003)	Cross-sectional	Survey	ETS was associated with significantly reduced lung function in asthmatic girls and nonsignificantly lower lung function in boys.
(Dinakar et al. 2005)	Cross-sectional	Biomarker (urinary cotinine)	ETS had no significant effect on ENO in asthmatic children
(Morkjaroenpong et al. 2002)	Cross-sectional	Survey on ETS exposure	Among inner-city children with asthma, exposure to moderate-to-heavy ETS was associated with increased frequency of nocturnal symptoms; a dichotomous ETS measure was not associated
(Spanier et al. 2006)	Cross-sectional	Survey on ETS and biomarkers (hair and serum cotinine)	No significant association of FeNO levels with ETS exposure by hair or serum cotinine, or several metrics of number of cigarettes smoked in home.
(Sturm et al. 2004)	Cross-sectional	Interview on ETS exposure	ETS exposure associated with significantly increased wheezing, with a dose-response relationship evident even at low levels of exposure (less than 1 day/month).
(Vargas et al. 2007)	Cross-sectional		No significant differences in acute asthma symptoms or response to emergency dept. therapy between ETS-exposed and unexposed children, but borderline significant association between ETS and pulmonary index.
(Wang et al. 2007)	Cross-sectional		Children with more than one emergency dept. visit reported more ETS at home than those with one such visit.

Source	Study Design	Measured Exposures	Findings
(Karadag et al. 2003)	Cross sectional	Urinary cotinine	The degree of passive tobacco smoke exposure was not higher during acute asthma attacks
Adults and Children			
(Herman and Walsh 2011)	Prospective	Natural intervention (ETS reduced by state smoking ban)	Statewide smoking ban decreased hospital admis-sionsfor asthma (note: outcome would include exacer-bations but also new asthma).
(Palmer et al. 2006)	Cross sectional	Survey	13- to 21-year-olds with the GSTM1-null genotype or homozygous for the GSTP1Val105 allele with envi-ronmental tobacco smoke exposures were more likely to have a substantially lower percentage of predicted peak expiratory flow rates than those from nonsmoking households
Adults			
(Eisner et al. 2002)	Prospective cohort	Survey (ETS in prior 7 days)	Higher ETS exposure at baseline associated with great-er asthma severity during follow-up (score based on frequency of current asthma symptoms, use of asthma medications, and history of hospitalizations and intuba-tions) and with greater healthcare utilization for asthma (emergency department visits and hospital admissions for asthma).
(Eisner et al. 2005)	Prospective cohort	Survey, personal nicotine badge, and hair analysis	Higher recent ETS exposure associated with greater asthma severity; ETS from previous month (nicotine badge) not associated with asthma severity; ETS from prior 2–3 months had only suggestion of association with greater asthma severity; ETS by nicotine badge not associated with hospital admissions; hair nicotine in prior month associated with greater risk of hospital admissions
(Newman et al. 2010)	Prospective cohort	Survey	Household ETS exposure associated nonsignificantly with less severe or frequent symptoms during pregnancy.

Abbreviations: ETS, environmental tobacco smoke; FeNO, fraction of inhaled nitric oxide; FEV_1, forced expiratory volume in 1 second; $FEV_{25–75\%}$, forced expiratory volume in mid 50% of exhaled breath; PEF, peak expiratory flow.

Table S6. Recent evidence on the association of dog allergens with exacerbation of asthma or asthma severity.

Source	Study Design	Measured Exposures	Findings
Children			
(McConnell et al. 2006)	Prospective	Dog presence	Significant dog × pollutant interaction effect on bronchitic symptoms. An increase in annual average ambient air pollution results in an increase in symptoms of bronchitis among asthmatic children; this effect occurs primarily among children with a dog in the home. Among children who owned a dog, an increase in the period prevalence of bronchitis was associated with this variability for all pollutants. The largest increase was for organic carbon (91% per 1.2 μg/m^3), followed by elemental carbon (74% per 0.29 μg/m^3). The smallest increase was for PM10–2.5 (30% per 4.2 μg/m^3).
(Gent et al. 2012)	Prospective	Dog allergen in dust from bedroom and living room	Significant positive association of dog allergen among sensitized with increased asthma severity
(Gent et al. 2009)	Prospective	Dog allergen in dust from bedroom and living room	Significant positive association of dog allergen with increased asthma severity among sensitized asthmatics
(Murray et al. 2006)	Cross-sectional (case-control)	Dog allergen in dust from bed and living room floor	Children in the acute exacerbation group were significantly more likely to be sensitized and exposed to dog allergen than those in the stable asthma
Adults			
(Langley et al. 2005)	Cross-sectional	Dog allergen in dust from living room	Atopic asthmatic subjects who are exposed to high levels of dog allergens but not sensitized to this allergens have significant evidence of increased airway reactivity.

Abbreviations: PM, particle mass.

Table S7. Recent evidence on association of fungi (quantified) with exacerbation of asthma or asthma severity.

**Source**	**Study Design**	**Measured Exposures**	**Findings**
Children			
(Pongracic et al. 2010)	Prospective cohort	Indoor and outdoor culturable airborne fungi levels (1-minute samples) measured at baseline and throughout the 2-year study	Among asthmatic children with any fungal sensitization, total indoor fungi, the four most common fungi combined (*Alternaria, Aspergillus, Cladosporium, and Penicillium*), and indoor *Penicillium* were associated with increased severe exacerbations (as UVs) after controlling for outdoor exposure; *Penicillium* exposure was associated with increased UVs among children who were fungally sensitized but not to *Penicillium*. Among species, only *Penicillium* exposure demonstrated significant effects among non-specifically sensitized. The sum of 4 most common indoor fungi and indoor *Penicillium* were associated with increased symptom days. In conclusion, outdoor and indoor fungi, especially *Penicillium* spp, worsen asthma morbidity in inner city children. Indoor *Penicillium* uniquely affected both symptoms and UVs.
Gent et al. 2012	Prospective (over 1 month)	Culturable airborne fungi (*Penicillium, Cladosporium)* (1-minute samples) in living room	Among specifically sensitized asthmatic children, relative to unexposed or exposed non-sensitive, significant positive association of any *Penicillium* exposure with doubled levels of increased asthma severity score, wheeze, and persistent cough. No associations seen with *Cladosporium*.
(Turyk et al. 2006)	Cross-sectional	Culturable airborne fungi (sampling time unspecified) in kitchen and bedroom	Bedroom *Penicillium,* in models adjusted for home dampness, had a significantly positive (dose-response) association with frequent asthma symptoms in asthmatic children (unknown atopic status), and a nearly significant positive association with number of asthma symptoms. No associations seen for kitchen *Penicillium*.
(Bundy et al. 2009)	Prospective (over 2 wks)	Culturable airborne *Penicillium* and *Cladosporium* (1-minute samples) in main living area of home	Any measured culturable airborne *Penicillium* in the main living area was significantly associated in asthmatic children (unknown sensitization, but adjusted for maternal-reported atopy) with more than doubled peak expiratory flow variability (PEFV) over next 2 weeks; exposure to any *Alternaria* was associated nonsignificantly with twice the likelihood to have the highest PEFV. No associations were found with total mold, *Cladosporium*, or *Aspergillus*. Analyses atopy-adjusted.
Inal et al. 2007	Prospective (monthly over 1 year)	Culturable airborne fungi (sampling time unspecified) in living room and bed room	In children with asthma (17 of 19) and/or rhinitis, sensitized only to molds, in unadjusted analyses in 19 children, neither total indoor molds, *Cladosporium, Alternaria, Penicillium, or Aspergillus* had significant correlations with daily asthma symptom score, morning PEF, or evening PEF.
(Wu et al. 2010)	Prospective cohort study (outcomes every 4 months over 4 years)	Total cultural fungi/in vacuumed dust from 5 home locations at initial visit, and genetic polymorphisms	For high values of total culturable fungi in house dust, although not directly related to outcomes of urgent care visits, lung function, IgE, or eosinophils, relationships with urgent care visits were significantly modified by three SNPs of chitinase, in unadjusted analyses. (As estimated from a figure, the association of high vs. low fungi with severe exacerbations (~30% vs. 0%) in those with no or one copy of SNP rs2486953 approximately doubled with two copies.). Thus, reduced enzymatic breakdown of fungal chitin may increase susceptibility to effects of chitin.

Abbreviations: PEFR, peak expiratory flow rate; FEV_1, forced expiratory volume in 1 second; IgE, Immunoglobulin E; PEFV, peak expiratory flow variability; SNPs, single nucleotide polymorphisms; UVs, unscheduled medical visits.

Table S8. Recent evidence on association of dampness, mold, or dampness-related agents with exacerbation of asthma.

Source	Study Design	Measured Exposures	Findings
Children			
(Kercsmar et al. 2006)	Randomized controlled intervention.	Remediation of root causes of home moisture and mold, removal of water damaged building materials, and cleaning	In the period after remediation, relative to the control group, visible mold scores were significantly more reduced (-2.6 vs.-1.4), and measured mold indices were nonsignificantly reduced (-0.41 vs. 0.33); in the remediation group compared to the control group, maximum symptom days were significantly reduced, and subjects having 1+ acute care visits were reduced by 64% in the as-randomized analysis, and by 86% in the as-treated analysis. About 1/3 of subjects were sensitive to fungi.
(Bernstein et al. 2006)	Controlled intervention	Intervention – 2-week ultraviolet radiation to reduce microbial exposures	In children sensitized to fungi, ultraviolet radiation was associated with a significant reduction in PEFR variability, and non-significant reduction in FEV_1; significant reductions in severity scores for shortness of breath and chest tightness, and in number of days of shortness of breath and chest tightness, as well as in amount of medication use; non-significant reductions in all other disease severity measures.
(Venn et al. 2003)	Prospective	Observed mold, and measured wall moisture	In children with persistent wheezing across a 3-year interval, dose-related increase in wheezing with increasing measured wall dampness, significantly more in atopic cases. For night time symptoms and bedroom dampness, OR 2.51 (1.36-4.64) per increasing category, with OR=7.0 for the highest category; for day-time symptoms and living room dampness, OR 1.86 (1.02-3.42) per increasing category. Visible mold was not significantly associated with either symptoms, although significantly associated with wheezing illness.
(Hagmolen of Ten Have et al. 2007)	Prospective	Parentally reported damp stains or mold growth, in living room or bedroom, in last 2 years	Damp stains or mold growth were associated with significant increases in severe airway hyperresponsiveness, more days with respiratory symptoms, and greater PEF variability.
(Bonner et al. 2006)	Cross-sectional	Presence of any moisture or mildew	Presence of moisture or mildew at home associated with 3.31 times more hospitalization visits for breathing-related problems, 3.25 times more frequent wheezing episodes, and expected 2.19 times greater frequency of night symptoms.
(Teach et al. 2006)	Cross sectional	Mold or dampness in the home in the previous month	Visible dampness or mold in the home in the previous month, in asthmatic children of unknown sensitization status, was not associated with unscheduled visits above the median, persistent asthma symptoms, or quality of life scores below the median.
Adults			
Williamson et al. 1997	Prospective	Inspector-assessed visible mold, and measured wall moisture as total dampness or worst dampness	In diagnosed asthmatics, asthma severity had significant positively dose-related association with measured total dampness and with visible mold score; measured airflow obstruction was significantly greater with higher measured dampness.
(Wen et al. 2009)	Cross-sectional	Visible mold from interview	Prevalence of asthma attacks among those exposed to indoor mold was roughly twice that in those not exposed, in either obese or non-obese subjects.

Abbreviations: ENO, exhaled nitric oxide; FEV_1; PEFR, peak expiratory flow rate; SNP, single nucleotide polymorphism; UVs, unscheduled medical visits.

Table S9. Recent evidence on association of nitrogen dioxide with exacerbation of asthma.

Source	Study Design	Measured Exposures	Findings
Children			
(Pilotto et al. 2004)	Randomized controlled inter-vention	NO_2	Asthma symptoms were reduced following an unflued gas heater replacement intervention in schools that removed high exposure to NO_2
(Gillespie-Bennett et al. 2011)	Controlled in-tervention	NO_2	In homes with or without unflued gas heater replacement, indoor NO_2 was associated with greater daily reports of lower and upper respiratory tract symptoms, more frequent coughing and wheezing and a reduction in morning and evening FEV_2.
(Belanger et al. 2013)	Prospective	NO_2	In asthmatic children, every 5-fold increase in NO_2 exposure above a threshold of 6 ppb was associated with a significant, dose-dependent increase in risk of higher asthma severity score (OR=1.4), wheeze (1.5), night symptoms (1.5), and rescue medication use (1.8). This was at levels well below the U.S. Environmental Protection Agency outdoor standard (53 ppb),
(Hansel et al. 2008)	Prospective	NO_2	Increasing NO_2 concentrations were significantly associated with increasing frequency of symptoms including limited speech due to wheezing; coughing without a cold; nocturnal awakenings due to cough, wheeze, and shortness of breath; and chest tightness during the daytime and while running, but were not associated with rescue medication use or unscheduled medical care visits (unscheduled doctor's visit, asthma-related hospital visit, or emergency department visit)
(Kattan et al. 2007)	Prospective	NO_2	NO_2 concentrations in the bedroom had a significant positive asso-ciation with days of wheeze among nonatopic children, but not among atopic children. There was no significant association with unscheduled emergency department or clinic visits. There was a significant inverse association in colder months with peak flow, but not in less cold months and not overall.
(Fu et al. 2012)	Prospective	NO_2	Children with high ADRB2 methylation exposed to high levels of NO_2 were 4.6 times more likely than children exposed to low levels to have severe asthma.
(Belanger et al. 2006)	Cross-sectional	NO_2	For asthmatic children in multifamily housing, measured NO_2 con-centrations had a significantly positive association with increased likelihood of wheeze and chest tightness and smaller nonsignificantly positive associations with persistent cough and shortness of breath. Findings for days per last month of each symptom were the same. In single family housing there were no associations of measured NO_2 with symptoms.
Adults			
(Ng et al. 2001)	Prospective	NO_2	In asthmatic women, acute short term exposure to NO_2 from single episodes of gas cooking was associated with immediate airflow limi-tation. Continued exposure from repeated episodes of gas cooking was associated with greater use of rescue bronchodilators.

Abbreviations: FEV_2, forced expiratory volume in 2 seconds.

Table S10. Recent evidence on association of gas stove use with exacerbation of asthma.

Source	Study Design	Measured Exposures	Findings
Children			
(Belanger et al. 2006)	Cross-sectional	Presence of gas stove in home	The presence of a gas stove in the home had a significant positive association with wheeze, shortness of breath, and chest tightness among asthmatic children living in multi-family housing, but not single-family housing.
(Chapman et al. 2003)	Cross-sectional	Gas stove, interview	There was a significant inverse association of use of a gas stove in the home for cooking on $FEF_{25–75\%}$, $FEF_{25–75\%}$ /FVC, FEV_1 and FEV_1/FVC among girls who were not taking prescription respiratory medication. No association among girls when respiratory medication was taken. No association among boys. Reported use of an exhaust fan had no impact on the effect of using a gas stove for cooking.
(Bonner et al. 2006)	Cross-sectional	Gas stove: whether gas stove has outside vented exhaust	Existence of a gas stove without outside vented exhaust was not associated with any measure of asthma severity, including wheezing (without a cold) during the prior 12 months, days with any asthma symptoms and nights awakened because of symptoms during the prior 14 days, school days missed per month enrolled, or the number of respiratory hospitalizations or emergency department visits in prior 12 months, attacks treated with oral steroids in prior four weeks.
Adults			
(Ng et al. 2001)	Prospective	NO_2	In asthmatic women, acute short term exposure to single episodes of gas cooking was associated with immediate airflow limitation. Continued exposure from repeated episodes of gas cooking was associated with greater use of rescue bronchodilators.
(Eisner et al. 2002)	Prospective	Personal use of gas stove for cooking	Personal use of a gas stove for cooking was not associated with an asthma severity score based on frequency of current asthma symptoms, use of systemic corticosteroids, use of other asthma medications, and history of hospitalizations and intubations, or with use of systemic corticosteroids, use of other asthma medications (besides systemic corticosteroids), and history of hospitalizations and intubations.
(Eisner and Blanc 2003)	Cross-sectional	Gas stove, interview	Gas stove use had no association with FEV_1, FVC, FEV_1/FVC ratio, or $FEF_{25–75\%}$; no association with chronic cough or phlegm production. Gas stove use was related to a greater risk of dyspnea, wheeze, and any respiratory symptom, but the 95% confidence intervals did not exclude no relationship.

Abbreviations: $FEF_{25–75\%}$, mid-flow forced expiratory flow (middle 50%); FEV_1, forced expiratory volume in 1 second; FVC, forced vital capacity.

Table S11. Recent evidence on association of formaldehyde (nonoccupational) with exacerbation of asthma.

Source	Study Design	Measured Exposures	Findings
Adults			
(Ezratty et al. 2007)	Double blind, randomized cross-over controlled exposure	60-minute exposures to formal-dehyde at mean concentrations of 500 µg/m^3 on 2 separate days	Exposure to 500 µg/m^3 formaldehyde had no significant deleterious effect on airway allergen responsiveness of patients with intermittent asthma
(Casset et al. 2006)	Double blind, randomized cross-over controlled exposure	30-minute exposures to formal-dehyde at mean concentrations of either 92.2 µg/m^3 (just below the World Health Organization's recommended maximum level) or (as baseline) 32.0 µg/m^3.	Formaldehyde exposure for 30 minutes did not cause changes in lung function and did not induce symptoms. It did, however, significantly enhance immediate and late airway responses to inhaled mite allergen in subjects with mild asthma. In short, in asthmatics, formaldehyde exposure increased effects of a common asthma trigger without having direct effect itself, and thus may increase allergic or non-allergic reactivity among asthmatics.

Table S12. Recent evidence on association of rodent allergen (nonoccupational) with exacerbation of asthma.

Source	Study Design	Measured Exposures	Findings
Children			
(Pongracic et al. 2008)	Randomized, unblinded environmental intervention with 2-year follow-up	Mouse allergen in dust from bedroom floor and mattress	Mouse allergen was prevalent in inner-city homes. Twenty-two percent of children tested positive to mouse. Sensitization occurred at low levels of exposure. Mouse allergen levels on the bedroom floor were reduced by 27.3% in intervention homes; bed allergen levels did not change for either group. Mouse allergen reduction was associated with less missed school, sleep disruption, and caretaker burden, but not symptoms or medical utilization. Mouse allergen was an independent risk factor for asthma morbidity.
(Phipatanakul et al. 2000)	Prospective	Mouse allergen in dust form bedroom, living room, and kitchen	Children whose homes had mouse allergen levels above the median (1.60 μg/g) in the kitchen had a significantly higher rate of mouse sensitization. The relationship among mouse allergen exposure, sensitization, and any measures of asthma morbidity was not statistically significant, although nonsignificant small increases (12-25%) were seen for three of eight morbidity outcomes.
(Bonner et al. 2006)	Cross-sectional	Presence of mouse or rat, from interview	Presence of mice or rats had no association with wheezing without a cold during the prior 12 months, days with any asthma symptoms, or nights awakened because of symptoms during the prior 14 days, or with hospitalizations or emergency department visits. No data on individual allergy status.

Table S13. Recent or additional evidence on association of endotoxin with exacerbation of asthma.

Source	Study Design	Measured Exposures	Findings
Children			
(Rabinovitch et al. 2005)	Cross-sectional	Endotoxins in personal air sampling with filters	Personal daily endotoxin exposures of children in $PM_{2.5}$ and PM_{10} particulate fractions were related to clinically significant increases in asthma severity indices. Personal endotoxin exposures had a significant positive association with asthma symptom scores and with evening FEV_1, but not with morning FEV_1.
Adults			
(Kitz et al. 2006)	Controlled inhalation challenge	Pre-post clinical trial spirometry (FEV1) and ENO, for LPS compared to saline	Asthmatic subjects experienced a significant fall in FEV_1 90 minutes after controlled inhalation challenge to LPS, reaching a maximum after 120 minutes. Overall, average ENO values were higher in non-sensitive asthmatics than in sensitive ones.
Adults and Children			
(Thorne et al. 2005)	Cross-sectional	Endotoxins in vacuumed house dust	Endotoxins in bedroom floor dust were associated with significantly elevated ORs for asthma symptoms, asthma medication use, and wheezing; similar but lower associations were found for bedding endotoxin concentrations and no associations were found with family room floor concentrations. No associations with increased risk of, or protection from, hay fever were found. These effects of endotoxin exposure were found in adults but not in children. Thus, exposures of adults, but not children, to household endotoxin were associated with asthma symptoms, current asthma medication use, and wheezing, but not with allergies.

Abbreviations: ENO, exhaled nitric oxide; FEV_1, forced expiratory volume in 1 second; LPS, lipopolysaccharide; OR, odds ratio; PM, particulate matter.

References

Belanger K, Gent JF, Triche EW, Bracken MB, Leaderer BP. 2006. Association of indoor nitrogen dioxide exposure with respiratory symptoms in children with asthma. Am J Respir Crit Care Med 173:297-303.

Belanger K, Holford TR, Gent JF, Hill ME, Kezik JM, Leaderer BP. 2013. Household levels of nitrogen dioxide and pediatric asthma severity. Epidemiology 24:320-330.

Bernstein JA, Bobbitt RC, Levin L, Floyd R, Crandall MS, Shalwitz RA, et al. 2006. Health effects of ultraviolet irradiation in asthmatic children's homes. J Asthma 43:255-262.

Bernton HS, McMahon TF, Brown H. 1972. Cockroach asthma. Br J Dis Chest. 66:61–66.

Bollinger ME, Eggleston PA, Flanagan E, Wood RA. 1996. Cat antigen in homes with and without cats may induce allergic symptoms. J Allergy Clin Immunol 97:907–914.

Bonner S, Matte TD, Fagan J, Andreopoulos E, Evans D. 2006. Self-reported moisture or mildew in the homes of head start children with asthma is associated with greater asthma morbidity. J Urban Health 83:129-137.

Bundy KW, Gent JF, Beckett W, Bracken MB, Belanger K, Triche E, et al. 2009. Household airborne penicillium associated with peak expiratory flow variability in asthmatic children. Ann Allergy Asthma Immunol 103:26-30.

California EPA (California Environmental Protection Agency). 1997. Health Effects of Exposure to Environmental Tobacco Smoke. Office of Environmental Health Hazard Assessment. Sacramento, CA.

Cantani A, Businco E, Maglio A. 1988. *Alternaria* allergy: a three-year controlled study in children treated with immunotherapy. Allergol Immunopathol (Madr) 16:1–4.

Casset A, Marchand C, Purohit A, le Calve S, Uring-Lambert B, Donnay C, et al. 2006. Inhaled formaldehyde exposure: Effect on bronchial response to mite allergen in sensitized asthma patients. Allergy 61:1344-1350.

Chapman RS, Hadden WC, Perlin SA. 2003. Influences of asthma and household environment on lung function in children and adolescents: The Third National Health and Nutrition Examination Survey. Am J Epidemiol 158:175-189.

Cockcroft DW, Ruffin RE, Frith PA, Cartier A, Juniper EF, Dolovich J, Hargreave FE. 1979. Determinants of allergen-induced asthma: dose of allergen, circulating IgE antibody concentration, and bronchial responsiveness to inhaled histamine. Am Rev Respir Dis 120:1053–1058.

Cook DG, Strachan DP. 1999. Health effects of passive smoke. 10. Summary of effects of parental smoking on the respiratory health of children and implications for research. Thorax 54:357-366.

Delfino RJ, Zeiger RS, Seltzer JM, Street DH, Matteucci RM, Anderson PR, Koutrakis P. 1997. The effect of outdoor fungal spore concentrations on daily asthma severity. Environ Health Perspect 105:622–635.

Dharmage S, Walters EH, Thien F, Bailey M, Raven J, Wharton C, et al. 2006. Encasement of bedding does not improve asthma in atopic adult asthmatics. Int Arch Allergy Immunol 139:132-138.

Dinakar C, Lapuente M, Barnes C, Garg U. 2005. Real-life environmental tobacco exposure does not affect exhaled nitric oxide levels in asthmatic children. J Asthma 42:113-118.

Dreborg S, Agrell B, Foucard T, Kjellman NI, Koivikko A, Nilsson S. 1986. A double-blind, multicenter immunotherapy trial in children, using a purified and standardized *Cladosporium herbarum* preparation. I. Clinical results. Allergy 41:131–140.

Dybendal T, Elsayed S. 1994. Dust from carpeted and smooth floors. VI. Allergens in homes compared with those in schools in Norway. Allergy 49:210–216.

Eisner MD, Yelin EH, Katz PP, Earnest G, Blanc PD. 2002. Exposure to indoor combustion and adult asthma outcomes: Environmental tobacco smoke, gas stoves, and woodsmoke. Thorax 57:973-978.

Eisner MD, Blanc PD. 2003. Gas stove use and respiratory health among adults with asthma in NHANES III. Occup Environ Med 60:759-764.

Eisner MD, Klein J, Hammond SK, Koren G, Lactao G, Iribarren C. 2005. Directly measured second hand smoke exposure and asthma health outcomes. Thorax 60:814-821.

El-Ghitany EM, Abd El-Salam MM. 2012. Environmental intervention for house dust mite control in childhood bronchial asthma. Environ Health Prev Med 17:377-384.

Eggleston PA, Rosenstreich D, Lynn H, Gergen P, Baker D, Kattan M, Mortimer KM, Mitchell H, Ownby D, Slavin R, Malveaux F. 1998. Relationship of indoor allergen exposure to skin test sensitivity in inner-city children with asthma. J Allergy Clin Immunol 102:563–570.

Ezratty V, Bonay M, Neukirch C, Orset-Guillossou G, Dehoux M, Koscielny S, et al. 2007. Effect of formaldehyde on asthmatic response to inhaled allergen challenge. Environ Health Perspect 115:210-214.

Fu A, Leaderer BP, Gent JF, Leaderer D, Zhu Y. 2012. An environmental epigenetic study of adrb2 5'-utr methylation and childhood asthma severity. Clin Exp Allergy 42:1575-1581.

Gelber LE, Seltzer LH, Bouzoukis JK, Pollart SM, Chapman MD, Platts-Mills TA. 1993. Sensitization and exposure to indoor allergens as risk factors for asthma among patients presenting to hospital. Am Rev Respir Dis 147:573–578.

Gent JF, Belanger K, Triche EW, Bracken MB, Beckett WS, Leaderer BP. 2009. Association of pediatric asthma severity with exposure to common household dust allergens. Environ Res 109:768-774.

Gent JF, Kezik JM, Hill ME, Tsai E, Li DW, Leaderer BP. 2012. Household mold and dust allergens: Exposure, sensitization and childhood asthma morbidity. Environ Res 118:86-93.

Gillespie-Bennett J, Pierse N, Wickens K, Crane J, Howden-Chapman P. 2011. The respiratory health effects of nitrogen dioxide in children with asthma. Eur Respir J 38:303-309.

Gold DR, Burge HA, Carey V, Milton DK, Platts-Mills T, Weiss ST. 1999. Predictors of repeated wheeze in the first year of life. The relative roles of cockroach, birth weight, acute lower respiratory illness, and maternal smoking. Am J Respir Crit Care Med 160:227–236.

Gruchalla RS, Pongracic J, Plaut M, Evans R, 3rd, Visness CM, Walter M, Crain EF, Kattan M, Morgan WJ, Steinbach S, Stout J, Malindzak G, Smartt E, Mitchell H. 2005. Inner City Asthma Study: relationships among sensitivity, allergen exposure, and asthma morbidity. J Allergy Clin Immunol. 115:478-85.

Hagmolen of Ten Have W, van den Berg NJ, van der Palen J, van Aalderen WM, Bindels PJ. 2007. Residential exposure to mould and dampness is associated with adverse respiratory health. British Society for Allergy and Clinical Immunology.37:1827-32.

Halken S, Host A, Niklassen U, Hansen LG, Nielsen F, Pedersen S, et al. 2003. Effect of mattress and pillow encasings on children with asthma and house dust mite allergy. J Allergy Clin Immunol 111:169-176.

Halonen M, Barbee RA, Lebowitz MD, Burrows B. 1982. An epidemiologic study of interrelationships of total serum immunoglobulin E, allergy skin-test reactivity, and eosinophilia. J Allergy Clin Immunol 69:221–228.

Hansel NN, Breysse PN, McCormack MC, Matsui EC, Curtin-Brosnan J, Williams DL, et al. 2008. A longitudinal study of indoor nitrogen dioxide levels and respiratory symptoms in inner-city children with asthma. Environ Health Perspect 116:1428-1432.

Hedlin G, Heilborn H, Lilja G, Norrlind K, Pegelow KO, Schou C, Lowenstein H. 1995. Long-term follow-up of patients treated with a three-year course of cat or dog immunotherapy. J Allergy Clin Immunol 96:879–885.

Herman PM, Walsh ME. 2011. Hospital admissions for acute myocardial infarction, angina, stroke, and asthma after implementation of arizona's comprehensive statewide smoking ban. Am J Public Health 101:491-496.

Hollander A, Doekes G, Heederik D. 1996. Cat and dog allergy and total IgE as risk factors of laboratory animal allergy. J Allergy Clin Immunol 98:545–554.

Horst M, Hejjaoui A, Horst V, Michel FB, Bousquet J. 1990. Double-blind, placebo-controlled rush immunotherapy with a standardized *Alternaria* extract. J Allergy Clin Immunol 85:460–472.

Inal A, Karakoc GB, Altintas DU, Guvenmez HK, Aka Y, Gelisken R, et al. 2007. Effect of indoor mold concentrations on daily symptom severity of children with asthma and/or rhinitis monosensitized to molds. J Asthma 44:543-546.

IOM. 2000. Clearing the air: Asthma and indoor air exposures. Washington, DC:National Academy Press. http://www.nap.edu/catalog.php?record_id=9610.

Kang B. 1976. Study on cockroach antigen as a probable causative agent in bronchial asthma. J Allergy Clin Immunol. 58:357–365.

Karadag B, Karakoc F, Ceran O, Ersu R, Inan S, Dagli E. 2003. Does passive smoke exposure trigger acute asthma attack in children? Allergol Immunopathol (Madr) 31:318-323.

Kattan M, Mitchell H, Eggleston P, Gergen P, Crain E, Redline S, Weiss K, Evans R III, Kaslow R, Kercsmar C, Leickly F, Malveaux F, Wedner HJ. 1997. Characteristics of inner-city children with asthma: the National Cooperative Inner-City Asthma Study. Pediatr Pulmonol 24:253–262.

Kattan M, Gergen PJ, Eggleston P, Visness CM, Mitchell HE. 2007. Health effects of indoor nitrogen dioxide and passive smoking on urban asthmatic children. J Allergy Clin Immunol 120:618-624.

Kercsmar CM, Dearborn DG, Schluchter M, Xue L, Kirchner HL, Sobolewski J, et al. 2006. Reduction in asthma morbidity in children as a result of home remediation aimed at moisture sources. Environ Health Perspect 114:1574-1580.

Kitz R, Rose MA, Borgmann A, Schubert R, Zielen S. 2006. Systemic and bronchial inflammation following lps inhalation in asthmatic and healthy subjects. J Endotoxin Res 12:367-374.

Kuehr J, Frischer T, Meinert R, Barth R, Forster J, Schraub S, Urbanek R, Karmaus W. 1994. Mite allergen exposure is a risk factor for the incidence of specific sensitization. J Allergy Clin Immunol 94:44–52.

Langley SJ, Goldthorpe S, Craven M, Woodcock A, Custovic A. 2005. Relationship between exposure to domestic allergens and bronchial hyperresponsiveness in non-sensitised, atopic asthmatic subjects. Thorax 60:17-21.

Lawson JA, Dosman JA, Rennie DC, Beach J, Newman SC, Senthilselvan A. 2011. Relationship of endotoxin and tobacco smoke exposure to wheeze and diurnal peak expiratory flow variability in children and adolescents. Respirology 16:332-339.

Lelong M, Henard J, Wattre P, Duprey J, Thelliez P, Miersman R. 1986. Does immediate-type respiratory allergy occur regarding *Stemphylium*? Evaluation of 39 challenge tests [French]. Allerg Immunol (Paris) 18:21, 23, 25–26.

Licorish K, Novey HS, Kozak P, Fairshter RD, Wilson AF. 1985. Role of *Alternaria* and *Penicillium* spores in the pathogenesis of asthma. J Allergy Clin Immunol 76:819–825.

Litonjua AA, Sparrow D, Weiss ST, O'Connor GT, Long AA, Ohman JL Jr. 1997. Sensitization to cat allergen is associated with asthma in older men and predicts new-onset airway hyperresponsiveness. The Normative Aging Study. Am J Respir Crit Care Med 156:23–27.

Malling HJ. 1986. Diagnosis and immunotherapy of mould allergy. IV. Relation between asthma symptoms, spore counts and diagnostic tests. Allergy 41:342–350

McConnell R, Berhane K, Molitor J, Gilliland F, Kunzli N, Thorne PS, et al. 2006. Dog ownership enhances symptomatic responses to air pollution in children with asthma. Environ Health Perspect 114:1910-1915.

Mohsenin V. 1987. Airway responses to nitrogen dioxide in asthmatic subjects. Journal of Toxicology and Environmental Health 22:371–380.

Morkjaroenpong V, Rand CS, Butz AM, Huss K, Eggleston P, Malveaux FJ, et al. 2002. Environmental tobacco smoke exposure and nocturnal symptoms among inner-city children with asthma. J Allergy Clin Immunol 110:147-153.

Murray CS, Poletti G, Kebadze T, Morris J, Woodcock A, Johnston SL, et al. 2006. Study of modifiable risk factors for asthma exacerbations: Virus infection and allergen exposure increase the risk of asthma hospital admissions in children. Thorax 61:376-382.

Newman RB, Momirova V, Dombrowski MP, Schatz M, Wise R, Landon M, Rouse DJ, Lindheimer M, Caritis SN, Sheffield J, Miodovnik M, Wapner RJ, Varner MW, O'Sullivan MJ, Conway DL.2010. The effect of active and passive household cigarette smoke exposure on pregnant women with asthma. Chest. 137:601-8.

Ng TP, Seet CS, Tan WC, Foo SC. 2001. Nitrogen dioxide exposure from domestic gas cooking and airway response in asthmatic women. Thorax 56:596-601.

Nitschke M, Pilotto LS, Attewell RG, Smith BJ, Pisaniello D, Martin J, et al. 2006. A cohort study of indoor nitrogen dioxide and house dust mite exposure in asthmatic children. J Occup Environ Med 2006: 48:462-469.

Norman PS, Ohman JL Jr, Long AA, Creticos PS, Gefter MA, Shaked Z, Wood RA, Eggleston PA, Hafner KB, Rao P, Lichtenstein LM, Jones NH, Nicodemus CF. 1996. Treatment of cat allergy with T-cell reactive peptides. American Journal of Respiratory and Critical Care Medicine 154:1623–1628.

Ostro BD, Lipsett MJ, Mann JM, Weiner M, Selner JS. 1994. Indoor air pollution and asthma: results from a panel study. Am J Respir Crit Care Med 149:1400–1406.

Palmer CN, Doney AS, Lee SP, Murrie I, Ismail T, Macgregor DF, et al. 2006. Glutathione s-transferase m1 and p1 genotype, passive smoking, and peak expiratory flow in asthma. Pediatrics 118:710-716.

Peat JK, Tovey E, Toelle BG, Haby MM, Gray EJ, Mahmic A, Woolcock AJ. 1996. House dust mite allergens. A major risk factor for childhood asthma in Australia. Am J Respir Crit Care Med 153:141–146.

Perzanowski MS, Divjan A, Mellins RB, Canfield SM, Rosa MJ, Chew GL, et al. 2010. Exhaled no among inner-city children in New York City. J Asthma 47:1015-1021.

Phipatanakul W, Eggleston PA, Wright EC, Wood RA. 2000. Mouse allergen. Ii. The relationship of mouse allergen exposure to mouse sensitization and asthma morbidity in inner-city children with asthma. J Allergy Clin Immunol 106:1075-1080.

Piacentini GL, Martinati L, Mingoni S, Boner AL. 1996. Influence of allergen avoidance on the eosinophil phase of airway inflammation in children with allergic asthma. J Allergy Clin Immunol 97:1079–1084.

Pilotto LS, Nitschke M, Smith BJ, Pisaniello D, Ruffin RE, McElroy HJ, et al. 2004. Randomized controlled trial of unflued gas heater replacement on respiratory health of asthmatic schoolchildren. Int J Epidemiol 33:208-214.

Pongracic JA, Visness CM, Gruchalla RS, Evans R, 3rd, Mitchell HE. 2008. Effect of mouse allergen and rodent environmental intervention on asthma in inner-city children. Ann Allergy Asthma Immunol 101:35-41.

Pongracic JA, O'Connor GT, Muilenberg ML, Vaughn B, Gold DR, Kattan M, et al. 2010. Differential effects of outdoor versus indoor fungal spores on asthma morbidity in inner-city children. J Allergy Clin Immunol 125:593-599.

Rabinovitch N, Liu AH, Zhang L, Rodes CE, Foarde K, Dutton SJ, et al. 2005. Importance of the personal endotoxin cloud in school-age children with asthma. J Allergy Clin Immunol 116:1053-1057.

Rabito FA, Carlson J, Holt EW, Iqbal S, James MA. 2011. Cockroach exposure independent of sensitization status and association with hospitalizations for asthma in inner-city children. Ann Allergy Asthma Immunol 106:103-109.

Rosenstreich DL, Eggleston P, Kattan M, Baker D, Slavin RG, Gergen P, Mitchell H, McNiff-Mortimer K, Lynn H, Ownby D, Malveaux F. 1997. The role of cockroach allergy and exposure to cockroach allergen in causing morbidity among inner-city children with asthma. N Engl J Med. 336:1356–1363.

Salome CM, Brown NJ, Marks GB, Woolcock AJ, Johnson GM, Nancarrow PC, Quigley S, Tiong J. 1996. Effect of nitrogen dioxide and other combustion products on asthmatic subjects in a home-like environment. Eur Respir 9:910–918.

Sarpong SB, Hamilton RG, Eggleston PA, Adkinson NF Jr. 1996. Socioeconomic status and race as risk factors for cockroach allergen exposure and sensitization in children with asthma. J Allergy Clin Immunol 97:1393–1401.

Sarpong SB, Han Y. 1999. A threshold of cockroach allergen (Bla g II) exposure and sensitization. American Journal of Respiratory and Critical Care Medicine 156:A128.

Sastre J, Ibanez MD, Lombardero M, Laso MT, Lehrer S. 1996. Allergy to cockroaches in patients with asthma and rhinitis in an urban area (Madrid). Allergy 51:582–586.

Schou C. 1993. Defining allergens of mammalian origin. Clin Exp Allergy 23:7–14.

Sears MR, Herbison GP, Holdaway MD, Hewitt CJ, Flannery EM, Silva PA. 1989. The relative risks of sensitivity to grass pollen, house dust mite, and cat dander in the development of childhood asthma. Clin Exp Allergy 19:419–424.

Shaver JR, Zangrilli JG, Cho SK, Cirelli RA, Pollice M, Hastie AT, Fish JE, Peters SP. 1997. Kinetics of the development and recovery of the lung from IgE- mediated inflammation: dissociation of pulmonary eosinophilia, lung injury, and eosinophil-active cytokines. Am J Respir Crit Care Med 155:442–448.

Shedd AD, Peters JI, Wood P, Inscore S, Forkner E, Smith B, et al. 2007. Impact of home environment characteristics on asthma quality of life and symptom scores. J Asthma 44:183-187.

Sicherer SH, Wood RA, Eggleston PA. 1997. Determinants of airway responses to cat allergen: comparison of environmental challenge to quantitative nasal and bronchial allergen challenge. J Allergy Clin Immunol 99:798–805.

Soussan D, Liard R, Zureik M, Touron D, Rogeaux Y, Neukirch F. 2003. Treatment compliance, passive smoking, and asthma control: A three year cohort study. Arch Dis Child 88:229-233.

Spanier AJ, Hornung R, Lierl M, Lanphear BP. 2006. Environmental exposures and exhaled nitric oxide in children with asthma. J Pediatr 149:220-226.

Spanier AJ, Hornung RW, Kahn RS, Lierl MB, Lanphear BP. 2008. Seasonal variation and environmental predictors of exhaled nitric oxide in children with asthma. Pediatr Pulmonol 43:576-583.

Spanier AJ, Kahn RS, Hornung RW, Wang N, Sun G, Lierl MB, et al. 2009. Environmental exposures, nitric oxide synthase genes, and exhaled nitric oxide in asthmatic children. Pediatr Pulmonol 44:812-819.

Sporik R, Holgate ST, Platts-Mills TA, Cogswell JJ. 1990. Exposure to house-dust mite allergen (*Der p* I) and the development of asthma in childhood. A prospective study. N Engl J Med 323:502–507.

Squillace SP, Sporik RB, Rakes G, Couture N, Lawrence A, Merriam S, Zhang J, Platts-Mills TA. 1997. Sensitization to dust mites as a dominant risk factor for asthma among adolescents living in central Virginia. Multiple regression analysis of a population-based study. Am J Respir Crit Care Med 156:1760–1764.

Sturm JJ, Yeatts K, Loomis D. 2004. Effects of tobacco smoke exposure on asthma prevalence and medical care use in North Carolina middle school children. Am J Public Health 94:308-313.

Swanson MC, Agarwal MK, Reed CE. 1985. An immunochemical approach to indoor aeroallergen quantitation with a new volumetric air sampler: studies with mite, roach, cat, mouse, and guinea pig antigens. J Allergy Clin Immunol 76:724–729.

Teach SJ, Crain EF, Quint DM, Hylan ML, Joseph JG. 2006. Indoor environmental exposures among children with asthma seen in an urban emergency department. Pediatrics 117:S152-158.

Thorne PS, Kulhankova K, Yin M, Cohn R, Arbes SJ, Jr., Zeldin DC. 2005. Endotoxin exposure is a risk factor for asthma: The national survey of endotoxin in united states housing. Am J Respir Crit Care Med 172:1371-1377.

Turyk M, Curtis L, Scheff P, Contraras A, Coover L, Hernandez E, et al. 2006. Environmental allergens and asthma morbidity in low-income children. J Asthma 43:453-457.

Utell MJ, Frampton MW, Roberts NJ Jr, Finkelstein JN, Cox C, Morrow PE. 1991. Mechanisms of nitrogen dioxide toxicity in humans. Res Rep Health Eff Inst 43:1–33.

U.S. DHHS (U.S. Department of Health and Human Services). 1984. The Health Consequences of Smoking: Chronic Obstructive Lung Disease. A Report of the Surgeon General. U.S. DHHS, Public Health Service, Office of the Assistant Secretary for Health, Office of Smoking and Health, Washington, DC. DHHS Pub. No. (PHS) 84-50205.

U.S. EPA (U.S. Environmental Protection Agency). 1992. Respiratory Health Effects of Passive Smoking: Lung Cancer and Other Disorders. EPA/600/6- 90/006F. Washington, DC.

Valovirta E, Koivikko A, Vanto T, Viander M, Ingeman L. 1984. Immunotherapy in allergy to dog: a double-blind clinical study. Ann Allergy 53:85–88.

Vanto T, Viander M, Koivikko A. 1980. Skin prick test in the diagnosis of dog dander allergy: a comparison of different extracts with clinical history, provocation tests and RAST. Clin Allergy 10:121–132.

Vanto T, Koivikko A. 1983. Dog hypersensitivity in asthmatic children. A clinical study with special reference to the relationship between the exposure to dogs and the occurrence of hypersensitivity symptoms. Acta Paediatr Scand 72:571–575.

van der Heide S, van Aalderen WM, Kauffman HF, Dubois AE, de Monchy JG. 1999. Clinical effects of air cleaners in homes of asthmatic children sensitized to pet allergens. . J Allergy Clin Immunol 104: 447–451.

Vargas PA, Brenner B, Clark S, Boudreaux ED, Camargo CA, Jr. 2007. Exposure to environmental tobacco smoke among children presenting to the emergency department with acute asthma: A multicenter study. Pediatr Pulmonol 42:646-655.

Venn AJ, Cooper M, Antoniak M, Laughlin C, Britton J, Lewis SA. 2003. Effects of volatile organic compounds, damp, and other environmental exposures in the home on wheezing illness in children. Thorax. 58:955-60.

Wang HC, McGeady SJ, Yousef E. 2007. Patient, home residence, and neighborhood characteristics in pediatric emergency department visits for asthma. J Asthma 44:95-98.

Warner JA. 1992. Environmental allergen exposure in homes and schools [editorial]. Clin Exp Immunol 22:1044–1045.

Wen XJ, Balluz L, Mokdad A. 2009. Do obese adults have a higher risk of asthma attack when exposed to indoor mold? A study based on the 2005 behavioral risk factor surveillance system. Public Health Rep 124:436-441.

Williamson IJ, Martin CJ, McGill G, Monie RD, Fennerty AG. 1997. Damp housing and asthma: a case-control study. Thorax. 52:229-34.

Wu AC, Lasky-Su J, Rogers CA, Klanderman BJ, Litonjua AA. 2010. Fungal exposure modulates the effect of polymorphisms of chitinases on emergency department visits and hospitalizations. Am J Respir Crit Care Med 182:884-889.

Zetterstrom O, Osterman K, Machado L, Johansson SG. 1981. Another smoking hazard: raised serum IgE concentration and increased risk of occupational allergy. Br Med J (Clin Res Ed) 283:1215– 1217.
